# Muscle Regeneration Can Be Rescued in a Telomerase Deficient Zebrafish Model of Ageing by MMP Inhibition

**DOI:** 10.1111/acel.70238

**Published:** 2025-09-25

**Authors:** Yue Yuan, Carlene Dyer, Robert D. Knight

**Affiliations:** ^1^ Centre for Craniofacial and Regenerative Biology King's College London, Guy's Hospital London London UK; ^2^ William Harvey Research Institute, Barts and the London School of Medicine and Dentistry Queen Mary University of London London UK

**Keywords:** ageing, cell behaviour, macrophage, muscle stem cell, regeneration, rejuvenation, satellite cell, zebrafish

## Abstract

Ageing progressively impairs skeletal muscle regeneration, contributing to reduced mobility and quality of life. While the molecular changes underlying muscle ageing have been well characterised, their impact on muscle stem cell (muSC) behaviour during regeneration remains poorly understood. Here, we leverage telomerase‐deficient *tert* mutant zebrafish larvae as an in vivo model of accelerated ageing to perform real‐time analysis of muSC dynamics following muscle injury. We demonstrate that the ageing‐like inflammatory environment in *tert* mutant disrupts muSC migration, impairs activation and proliferation, and compromises regenerative capacity. We further show that sustained inflammation, mediated by persistent macrophage presence and elevated matrix metalloproteinase (MMP) activity, limits muSC recruitment and migration efficiency. Pharmacological inhibition of MMP9/13 activity and genetic depletion of macrophages partially restore muSC migratory behaviour and regenerative outcomes. Notably, we demonstrate that muSC migration dynamics correlate with regenerative success, providing a functional readout for therapeutic screening. Our findings reveal zebrafish *tert* mutants offer a tractable system for dissecting age‐associated changes to cell behaviour and for identifying rejuvenation interventions.

## Graphical abstract

Inhibition of MMP9/13 restores both muSC behaviour and regeneration, while macrophage ablation enhances muSC recruitment to the injury, but fails to improve muscle repair in telomerase‐deficient zebrafish larvae.

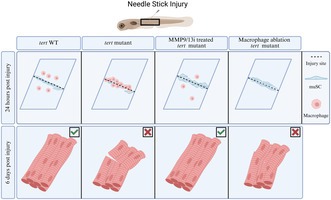

## 1 Introduction

Skeletal muscle regeneration declines significantly with age, leading to impaired repair and progressive loss of muscle mass and function. This regenerative failure arises from both intrinsic muSC dysfunction and extrinsic changes in the tissue microenvironment (Brack and Muñoz‐Cánoves 2016; Henze et al. 2020). In young muscle, injury triggers a well‐orchestrated response in which muSC activate, proliferate and differentiate into new myofibres, while immune cells facilitate repair through transient inflammatory signalling. However, ageing disrupts this balance. Aged muSCs exhibit reduced proliferative capacity and increased susceptibility to senescence, driven by chronic activation of stress pathways such as p38 MAPK signalling (Muñoz‐Cánoves et al. 2020). Although p38 MAPK activity is crucial for muSC differentiation under normal conditions, its persistent activation in aged tissues contributes to impaired regeneration by prematurely limiting muSC proliferation and self‐renewal. This cell‐intrinsic deterioration is compounded by changes in the aged muscle niche, where chronic low‐grade inflammation (‘inflammaging’) leads to prolonged exposure to inflammatory cytokines such as IL‐6 and TNFα. Overactivation of NF‐κB signalling, a key transcriptional regulator of pro‐inflammatory pathways, is also implicated in various age‐related diseases, including sarcopenia (Cornish and Cordingley 2024). This inflammatory persistence drives muSC exhaustion and impairs differentiation, contributing to fibrotic tissue remodeling and inefficient muscle repair (Bouché et al. 2014).

Although a number of studies have examined the impact of ageing on muSC function, we know little about how ageing affects muSC behaviour during regeneration (Brack and Muñoz‐Cánoves 2016). In particular, it remains unclear whether aged muSCs show any changes to their migratory behaviour, or have altered interactions with their environment and other cell types, such as macrophages, that are crucial for orchestrating repair. To investigate how ageing affects muSC function, we have evaluated muSC function in telomerase‐deficient (*tert* mutant) zebrafish ( *Danio rerio* ) (Pipalia et al. 2023). *Tert* mutant zebrafish are a model for premature ageing showing muscle atrophy, chronic inflammation and impaired regenerative capacity (Henriques et al. 2013). F2 larvae generated from inbreeding of telomerase mutant animals show increased cellular senescence, elevated apoptosis and tissue degeneration indicative of an accelerated ageing phenotype (Hernández‐Silva et al. 2025). Although *tert* mutants do not show all of the systemic metabolic and endocrine changes seen in ageing mammals, they do show a similar persistent inflammatory state and elevation of p53 signalling (Carneiro et al. 2016).

We find key hallmarks of ageing during muscle repair in *tert* mutants, involving delayed regeneration of muscle fibres, reduced muSC proliferation and persistent macrophage infiltration following injury. Live imaging reveals impaired muSC activation and migration in *tert* mutants is related to an aberrant inflammatory cell response. We demonstrate that inhibition of MMP9/13 can rescue muSC behaviour and regenerative capacity and is related to immune cell‐dependent remodelling of the extracellular matrix. In summary, we show that telomerase‐deficient zebrafish are a powerful platform for identifying molecules driving aberrant cell function and behaviour in ageing.

## 2 Materials and Methods

### 2.1 Fish Stocks and Husbandry

Animals were reared at the Kings College London Zebrafish Facility and maintained in accordance with UK Home Office regulation, UK Animals (Scientific Procedures) Act 1986, under project licence PP9727122. Fish carrying allele *tert* ^*sa6541*^ were obtained from Dr. Elisabeth Busch‐Nentwich from Queen Mary University of London and maintained in the AB background. Animals were in‐crossed to generate *tert* ^*sa654/1sa6541*^ mutants (*tert* mutants) or wildtype (WT) animals. Transgenic lines TgBAC[pax7a:egfp] (pax7a:egfp) (Mahalwar et al. 2014) and TgBAC[fms:gal4; UAS:NfsB‐mCherry] (fms:mCherry) (Gray et al. 2011) were crossed against animals carrying the *tert* ^*sa65421*^ allele for visualisation of muSCs and macrophages. Embryos were obtained from natural spawning, and embryonic fish were maintained in E3 Phenylthiourea (PTU) solution at 28.5°C to inhibit pigment formation medium (Westerfield 2000).

### 2.2 Genotyping

DNA from embryos or fin biopsies was extracted and genotyped for *tert* mutants using KASP genotyping as previously described (Cuppen 2007).

### 2.3 Needlestick Injury

Larvae were anesthetised in 0.004% w/v Tricaine (Sigma‐200 mg/mL) in E3 media and mounted in 1.5% w/v low melt agarose (Sigma). To induce muscle injury, a sharpened tungsten wire was applied to the myotome as previously described (Sultan et al. 2021).

### 2.4 Immunolabelling and BrdU Incorporation

The detection of Pax7 was performed as previously described (Roy et al. 2017) using mouse anti‐Pax7 (developed by A. Kawakami at the Tokyo Institute of Technology, obtained from the Developmental Studies Hybridoma Bank (DSHB), created by the NICHD of the NIH and maintained at The University of Iowa, Department of Biology, Iowa City, IA, United States).

Primary antibodies used included chick anti‐GFP (ab13970, Abcam) and SV2 supernatant (Q7L0J3, DSHB). Acetylcholine receptors were detected using alpha Bungarotoxin CF555 (00018‐100 μg, Insight Biotechnology).

For BrdU labelling larvae were exposed to 10 mM BrdU in the medium, then fixed with 4% PFA. Antibody detection involved treatment with 2 M HCL for 1 h at RT, neutralisation in 0.1 M borate buffer (0.62 g Boric acid, 1.5 mL 75 mM NaCl in 100 mL water; pH 8.5), then washed with PBT containing 1% v/v Triton X‐100 and 1% v/v DMSO. Primary antibody used was rat anti‐BrdU (ab6326, Abcam).

### 2.5 Whole‐Mount Immune‐Coupled Hybridisation Chain Reaction (WICHCR)

WICHCR were carried out as described previously (Ibarra‐García‐Padilla et al. 2021). Probes for *mmp9* were designed and purchased from Molecular Instruments.

### 2.6 RNA Isolation and Quantitative RT‐PCR

Total RNA was isolated from dissected injured trunk regions of larvae at 24 h post injury (hpi). Tissue was homogenised in TriReagent (Sigma) and total RNA isolated according to the manufacturer's protocol. 2 μg of total RNA was reverse transcribed into cDNA using random hexamer primers (Promega) and M‐MLV reverse transcriptase (Promega).

Quantitative RT‐PCR was performed on a C1000 TM Thermal Cycler with CFX384 Optical Reaction Module (Bio‐Rad) using TaqMan Gene Expression Assays (Thermo Fisher Scientific). Expression data were calculated according to the ΔΔCt method with 18S RNA as an internal expression control.

### 2.7 RNA Sequencing and Analysis

RNA analyses were performed on muscle dissected from 30 pooled larvae for each condition, or from individual 18‐month *tert* mutant and heterozygous animals (Table S1). mRNA was purified from total RNA using poly‐T oligo‐attached magnetic beads. After fragmentation, the first strand cDNA was synthesised using random hexamer primers, followed by the second strand cDNA synthesis using dTTP for non‐directional library. Index of the reference genome was built using Hisat2 v2.0.5 and paired‐end clean reads were aligned to the reference genome Danio_rerio.GRCz11.dna using Hisat2 v2.0.5. Differential expression analysis was performed using the DESeq2 R package (v1.40.2). Genes with low counts (sum across all samples ≤ 10) were excluded to reduce noise from low‐expression transcripts. The resulting *p*‐values were adjusted for multiple testing using the Benjamini‐Hochberg method, yielding a false discovery rate (FDR). Differentially expressed genes were defined as those with an adjusted *p*‐value (FDR) < 0.05. Gene Ontology (GO) enrichment analysis of differentially expressed genes was implemented by the ClusterProfiler R package, in which gene length bias was corrected.

### 2.8 Treatment of Larvae With Chemicals

MMP9/13 inhibitor I (15942, Cayman Chemical) was reconstituted to a stock concentration of 20 mM in fresh DMSO, aliquoted and stored at −80°C. The stock solution was then diluted to a working concentration in medium at a concentration of 100 μM.

Macrophage ablation was achieved by treating fms:mCherry larvae with 5 mM metronidazole (MTZ, Sigma) in media 24 h prior to injury. Following injury, larvae were placed into fresh 5 mM MTZ for the duration of the experiment. MTZ solution was refreshed every 12–14 h.

### 2.9 Imaging Acquisition

Images of fixed samples were acquired using a Carl Zeiss LSM980 using a 20× air objective (NA 0.8). Z‐stacks encompassing the total myotome (with 1 μm Z‐slices) were captured. Z‐stacks were captured at a resolution of 1024 × 1024 pixels, and each channel was averaged 4 times. For live imaging, larvae were anesthetised in 0.004% w/v Tricaine and mounted in 1.5% w/v low melt agarose in medium on a glass bottom dish with size 0 glass (IBL). Time‐lapsed images were captured every 15 min from 5 to 24 h post injury (hpi).

### 2.10 Image Processing, Cell Counting and Analysis

For cell counting, images were processed using Fiji (Schindelin et al. 2012). Brightness and contrast were adjusted to identify eGFP, BrdU and Pax7 expressing cells. Cells were manually counted from z‐stacks.

For processing of live imaging data, Z‐projections were generated by maximum intensity projection and drift was corrected using the Fast4Dreg Plugin (Laine et al. 2019); the image was then manually cropped for cell segmentation. GFP positive cells were segmented using cellpose2 (Pachitariu and Stringer 2022) with a custom trained model and cells tracked in Imaris 10.2 (Bitplane AG) using the Cells function with manual correction where necessary. Due to variations in the fluorescence intensity between samples, the threshold values were manually chosen for each individual sample to differentiate between visible cells and background noise. Following thresholding, the tracking algorithm was chosen to track the cells using the following parameters: max distance = 5.00 μm, max gap size = 2 μm and track duration above 2.5 s.

Parameters of cell movement were exported for statistical analysis: distance from origin [μm], circularity, mean square displacement [μm^2^] and speed mean [μm/s].

Neuromuscular junction (NMJ) structural integrity analysis was performed using NMJ Analyser as described by the authors (Singh et al. 2023).

### 2.11 Statistical Analysis

Statistical analysis was performed using GraphPad Prism Version 10.4.1. Measures of cell numbers acquired from samples processed by immunolabelling were tested for a normal distribution using a Kolmogorov–Smirnov test. Significance was analysed using Student *t*‐test. All *p*‐values are indicated in the figures (ns, not significant; *p < 0.05; **p < 0.01; ***p < 0.001; ****p < 0.0001).

Multiple linear regression models with mixed effects were generated to test how mean square displacement (MSD) was affected by genotype or treatments in a time dependent manner using the Stata v15 package (StataCorp) (Tables S2–S4).

## 3 Result

### 3.1 A Zebrafish *tert* Mutant Model Reveals Molecular and Functional Changes in Zebrafish Larvae Muscle

**FIGURE 1:**
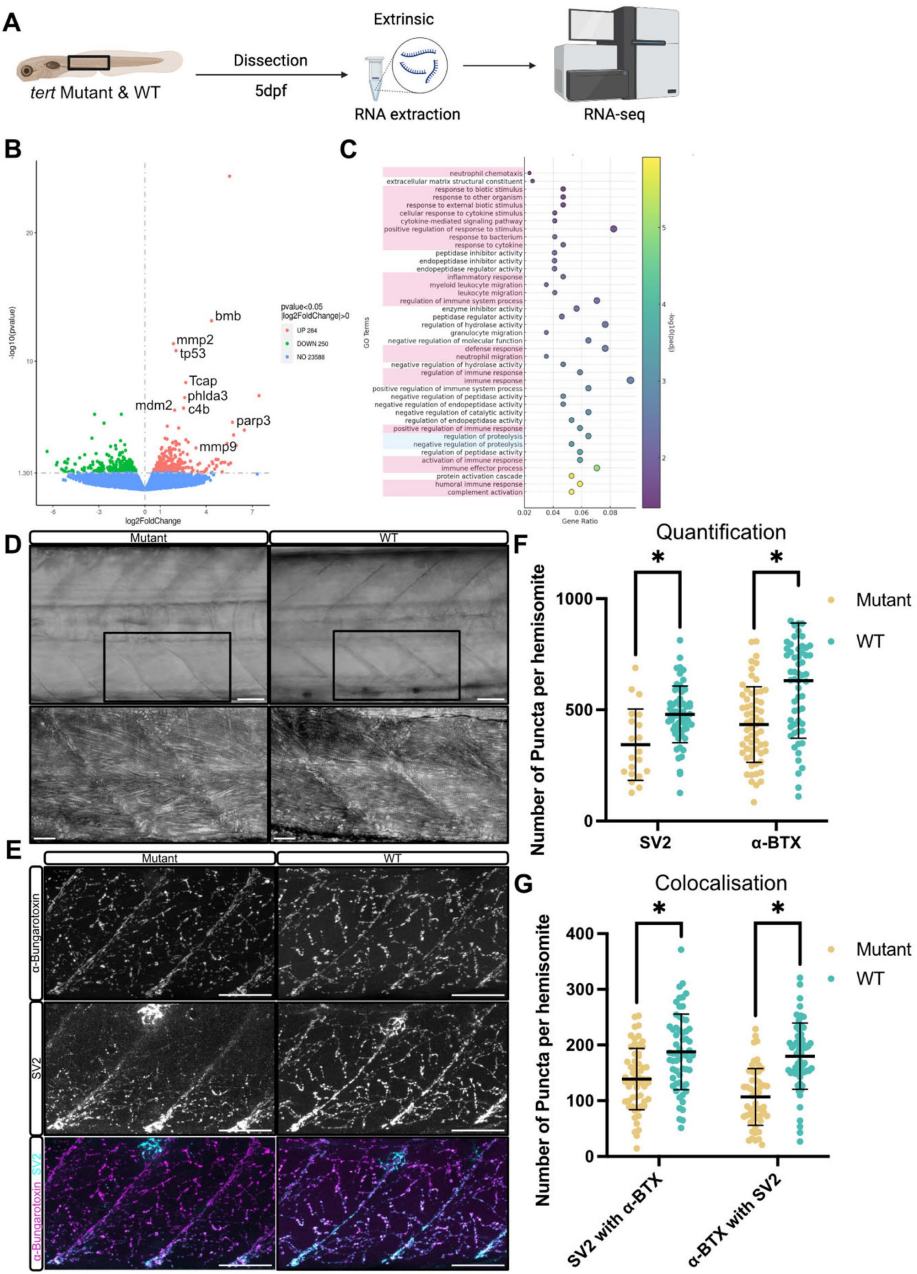
Muscle of zebrafish *tert* mutant larvae shows anatomical and molecular changes reminiscent of ageing. (A) Schematic representation of the experimental workflow. Muscle tissue was dissected from *tert* mutant and WT zebrafish larvae at 5 dpf, followed by RNA extraction and RNA‐seq analysis. (B) Volcano plot of differentially expressed genes in *tert* mutant muscle compared to WT controls. Upregulated genes involved in inflammation (*tp53*, *bmb*), DNA damage (*tp53*, *parp3*) and extracellular matrix (ECM) remodelling (*mmp2*, *mmp9*) are highlighted. (C) Gene ontology (GO) enrichment analysis of differentially expressed genes in *tert* mutant muscle. Terms related to inflammation and stress response are highlighted in pink, and loss of proteostasis is highlighted in blue. (D) Brightfield images of muscle morphology in WT and *tert* mutant larvae. (E) Representative immunofluorescence images of NMJs labelled with α‐bungarotoxin (AChR clusters, cyan) and synaptic vesicle protein SV2 (presynaptic marker, white). (F) Quantification of SV2 and α‐bungarotoxin puncta in ventral myotomes of *tert* mutants and WT siblings. (G) Quantification of colocalised puncta between α‐bungarotoxin and SV2. Number of animals used *n* = 8 (*tert* mutant), *n* = 7 (WT). Data are presented as mean ± SD, and statistical significance was determined using an unpaired Student's *t*‐test (*p < 0.05). Scale bars: 50 μm.

To understand how muscle is affected by loss of telomerase function in zebrafish, we profiled gene expression changes in dissected muscle from 5 days post‐fertilisation (5 dpf) *tert* larvae by RNA sequencing (RNA‐seq) (Figure 1A). Differential gene expression and Gene Ontology (GO) analyses revealed significant changes in genes related to inflammation, matrix organisation, cellular stress and proteostasis (Figure 1B,C, Table S1). Prominent inflammatory markers such as *mmp9*, *cxcl18b* and *tnfrsf1a* were up‐regulated, indicative of an elevated inflammatory state.

Histological examination of muscle tissue revealed no obvious morphological differences between *tert* mutant and WT larvae. Muscle fibres in both genotypes appeared structurally intact and well‐aligned (Figure 1D). However, neuromuscular junction (NMJ) integrity, assessed through immunofluorescent staining of presynaptic marker SV2 and postsynaptic acetylcholine receptors (AChR, labelled with α‐bungarotoxin), was compromised in *tert* mutants, with both a reduced number of individual presynaptic and postsynaptic puncta and co‐localisation between them (Figure 1E–G). In addition, we observed a reduced proliferation of muSCs using BrdU incorporation assays, suggesting impaired regenerative potential in *tert* mutant larvae (Figure S1).

To further assess the relevance of the *tert* mutant larval dataset in modelling age‐associated muscle dysfunction, we performed RNA‐seq analysis on skeletal muscle from 18‐month‐old *tert* mutants and their heterozygous siblings (Figure S2A). GO enrichment analysis revealed upregulation of pathways consistent with hallmarks of muscle ageing, including impaired muscle structural organisation and differentiation, chronic inflammation and defective regenerative signalling, DNA damage response, ECM remodelling and disrupted mitochondrial function (Figure S2B–F). Differentially expressed genes in mutant adult and larval staged animals included genes previously found to be involved in sarcomere integrity (*tcap*) (Zhang et al. 2009), cell stress and immune modulation (*c4b*, *eva1bb*, *vsig8a*, *trpm1b* and *btr24*) (Lin et al. 2010; Ricklin et al. 2010), ECM modification (*mmp2*) (Freitas‐Rodríguez et al. 2017) and DNA damage responses (*tp53*, *phlda3*, *mdm2* and *parp3*) (Brady et al. 2011). These findings support the notion that ageing phenotypes such as inflammatory signalling and ECM dysregulation are evident at larval stages in *tert* mutants.

### 3.2 Loss of Tert Function Impairs Muscle Stem Cell Activation, Proliferation and Regeneration

**FIGURE 2:**
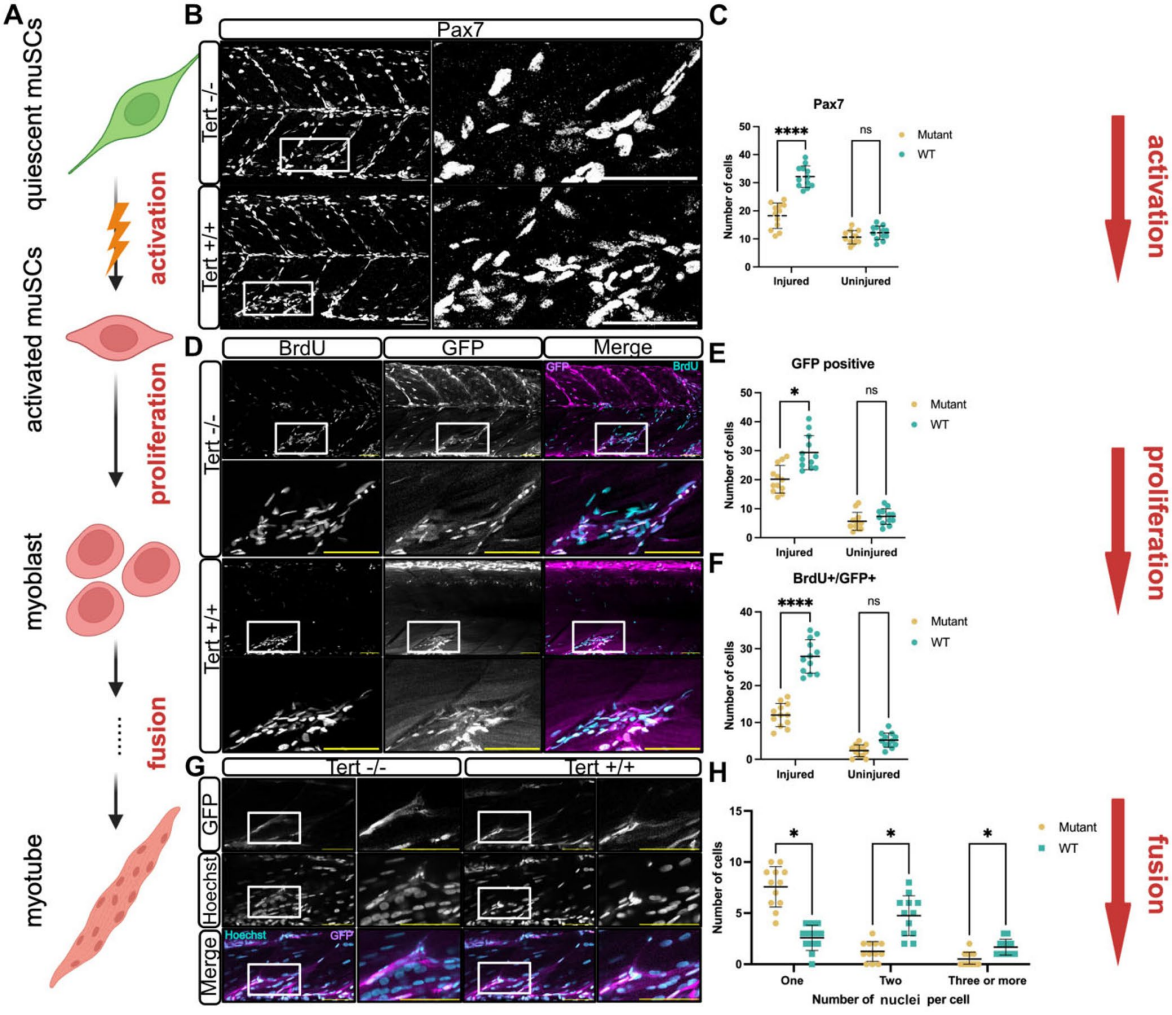
Loss of tert impairs muSC activation, proliferation and regeneration. (A) Schematic representation of muSC activation, proliferation and fusion process following injury. Quiescent muSCs become activated, proliferate, and ultimately fuse to form multinucleated myofibres during regeneration. (B) Representative images of Pax7 antibody labelling in WT and *tert* mutant larvae at 24 hpi. (C) Quantification of Pax7^+^ cells in *tert* mutant and WT. Number of animals used *n* = 12 (*tert* mutant), *n* = 12 (WT). (D) Representative images showing BrdU incorporation (cyan) and GFP expression (magenta) in larvae expressing a pax7a:egfp transgene at 24 hpi. (E, F) Quantification of GFP and BrdU/GFP double‐positive cells in *tert* mutant larvae and WT following injury. Number of animals used *n* = 12 (*tert* mutant), *n* = 12 (WT). (G) Representative images of regenerating muscle at 96 hpi, showing multinucleated myofibres in WT and *tert* mutant larvae. Hoechst staining (blue) marks nuclei. (H) Quantification of multinucleated myofibres. Number of animals used *n* = 12 (*tert* mutant), *n* = 12 (WT). Data are presented as mean ± SD, and statistical significance was determined using an unpaired Student's *t*‐test (ns, not significant, *p < 0.05, ****p < 0.0001). Scale bars: 50 μm.

We wished to know how the molecular changes observed in *tert* mutants related to muSC function (Figure 2A). We had noted there is a deficit in muSC proliferation under homeostatic conditions and hypothesised this would correspond to an impaired regenerative response, as described in mice. We found there were significantly fewer Pax7^+^ muSCs responding to injury in *tert* mutants compared to WT (Figure 2B,C). Using a pax7a:egfp transgene to identify muSC‐derived cells, we showed BrdU incorporation in muSCs was reduced compared to WT larvae at 24 h post injury (hpi) in *tert* mutants (Figure 2D–F). Importantly, this difference was not attributed to an increased cell death as acridine orange staining did not reveal discernible differences in cell death relative to WT larvae (Figure S3).

To understand whether regeneration of fusion of myoblasts was affected in *tert* mutants, we quantified nuclear number within newly formed GFP positive myofibres. Quantification of myofibre nuclear number revealed fewer multinucleated muscle fibres in *tert* mutant larvae at 96 hpi, indicating a reduced rate of myoblast fusion (Figure 2G–H). Collectively, these findings reveal that an impaired response by muSCs to injury occurs in *tert* mutants, resulting in attenuated regeneration of muscle.

### 3.3 muSC Behaviour During Regeneration Is Disrupted in *tert* Mutants

**FIGURE 3:**
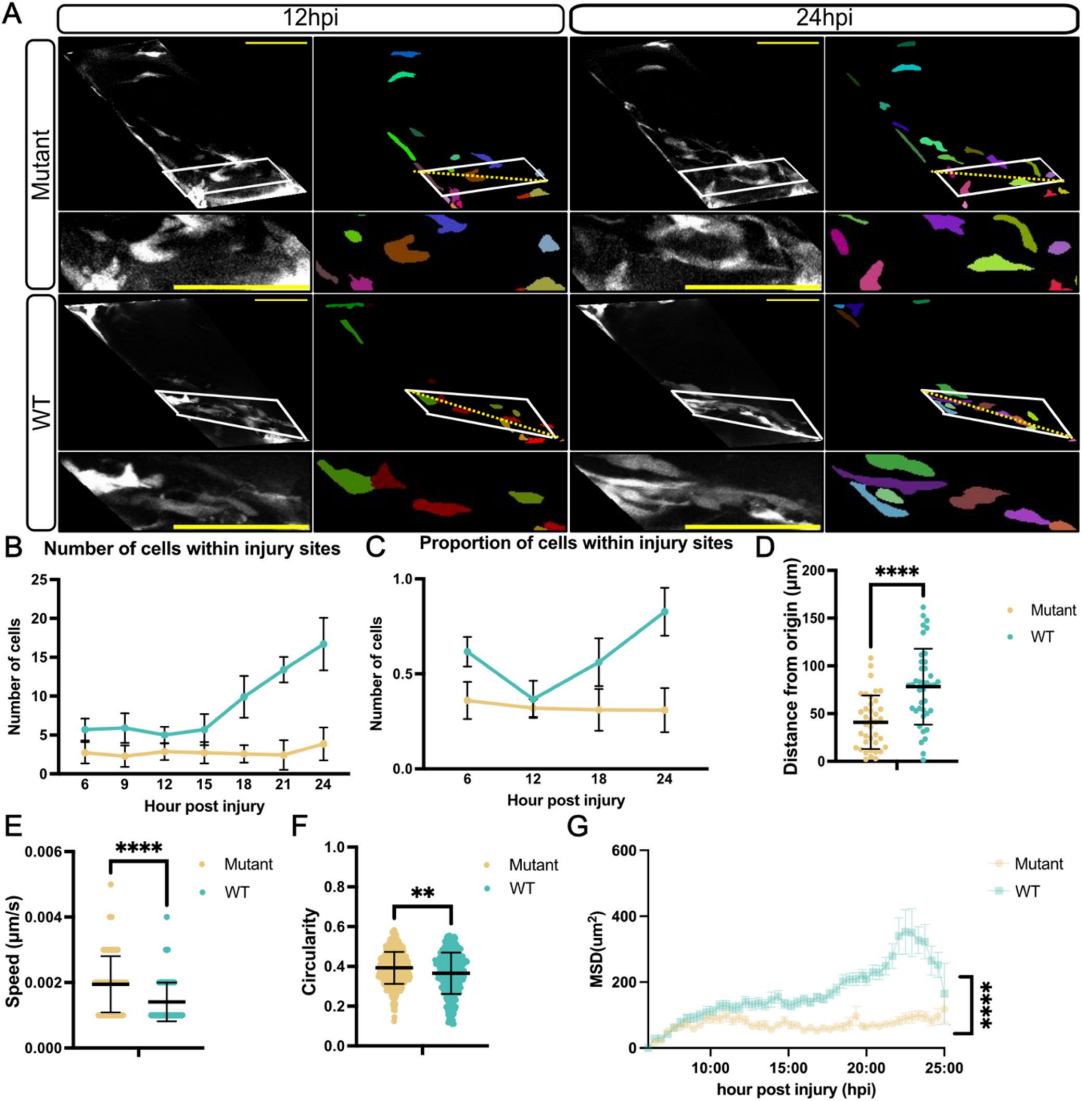
muSC migration is altered in *tert* mutants. (A) Images of time‐lapsed movies of muscle from 6 hpi to 24 hpi in WT and *tert* mutant larvae expressing pax7a:egfp. Lower panels show segmented cells (coloured). Yellow dashed line indicates the injury site. Quantitative analyses show (B) the number of muSCs within injury sites, (C) the proportion of cells within the injury sites relative to total muSC numbers in the injured myotome, (D) distances travelled from origin, (E) mean cell speed, (F) cell circularity and (G) mean squared displacement (MSD). Number of animals used *n* = 7 (*tert* mutant), *n* = 10 (WT). Data are presented as mean ± SD and statistical significance was determined using an unpaired Student's *t*‐test and linear regression model test for MSD (**p < 0.01, ****p < 0.0001). Scale bars: 50 μm.

Given the critical role of muSC migration for muscle regeneration, we asked whether changes to the capacity of muSCs to respond to injury in *tert* mutants corresponded with an altered migratory behaviour. Using live imaging of larvae expressing the pax7a:egfp transgene, we tracked muSC movements from 6 hpi to 24 hpi in both *tert* mutant and WT larvae (Figure 3A, Movies S1 and S2). Quantification of muSC localisation and movement revealed striking differences between WT and *tert* mutant larvae (Figure 3A). In WT larvae, muSCs migrated efficiently toward injury sites and accumulated progressively, becoming aligned with adjacent fibers at the injury site by 24 hpi. Conversely, muSCs in *tert* mutants exhibited a reduced and restricted migration, with fewer cells present at the injury over time (Figure 3A–C).

Further detailed analysis of cell movement and cell shape revealed that although mean cell speed was increased, muSCs moved significantly shorter distances from their origin and showed greater circularity, indicative of fast yet confined movements (Figure 3D–F). Multiple linear regression models were used to test whether the mean squared displacement (MSD) showed significant differences between *tert* mutant and WT. MSD was significantly lower in *tert* mutants than in WT animals, indicative of impaired cell migration (Figure 3G, Table S2).

Our characterisation of cell response to injury reveals an abnormal migratory response by muSCs in *tert* mutants, characterised by an increased speed but showing reduced directional movement, resulting in fewer cells present within the injury at 24 hpi.

### 3.4 Injured *tert* Mutants Exhibit Prolonged Inflammation and Delayed Macrophage Clearance Following Injury

**FIGURE 4:**
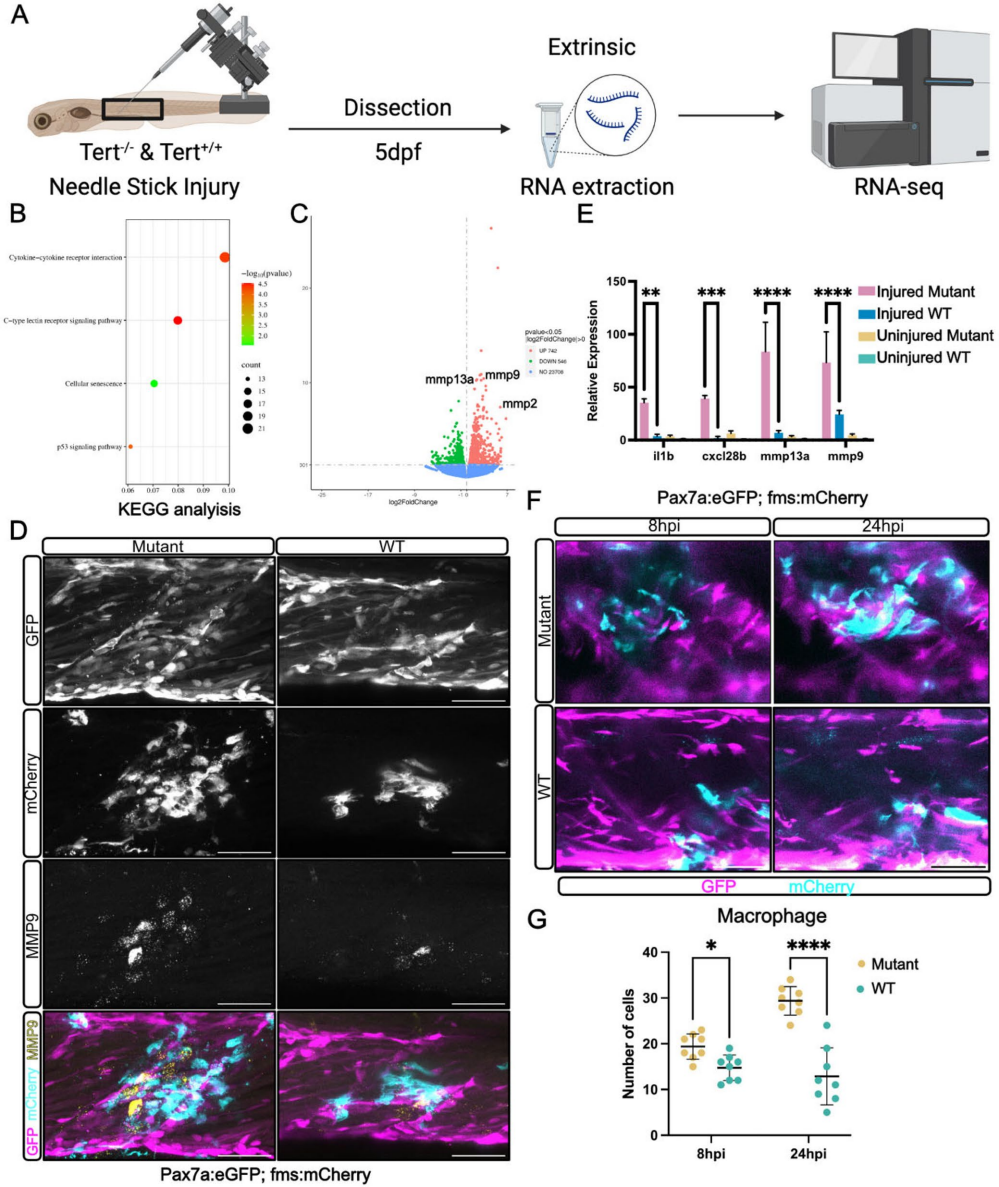
Injured *tert* mutant shows prolonged inflammatory response and delayed macrophage clearance following injury. (A) Schematic representation of the experimental workflow. Muscle from injured *tert* mutant and WT zebrafish larvae was extracted and profiled by RNA‐seq. (B) KEGG pathway analysis of differentially expressed genes in *tert* mutant versus WT injured muscle. (C) Volcano plot showing upregulation of *mmp2*, *mmp9* and *mmp13* in *tert* mutants. (D) Representative images of HCR detection of *mmp9* expression relative to muSCs expressing pax7a:egfp (GFP, magenta) and macrophages expressing fms:mCherry (mCherry, cyan); number of animals *n* = 6 (*tert* mutant), *n* = 6 (WT). (E) qPCR measures of inflammatory (*il1b*, *cxcl28b*) and *mmp13a*, *mmp9* gene expression. (F) Representative images of muSCs expressing pax7a:egfp (GFP, magenta) and macrophages expressing fms:mCherry (mCherry, cyan) in injured muscle of *tert* mutant and WT larvae at 8 hpi and 24 hpi. (G) Quantification of macrophages in *tert* mutants and WT larvae at 8 hpi and 24 hpi, number of animals used *n* = 10 (*tert* mutant), *n* = 10 (WT). Data are presented as mean ± SD, and statistical significance was determined using an unpaired Student's *t*‐test (*p < 0.05, **p < 0.01, ***p < 0.001, ****p < 0.0001). Scale bars: 50 μm.

To understand whether *tert* mutants show an altered response to injury that affects muSC responses, we performed RNA‐seq on injured muscle tissue of 5 dpf larvae at 24 h after needle‐stick injury (Figure 4A). KEGG pathway analysis revealed significant upregulation of inflammatory pathways in *tert* mutants compared to WT larvae, particularly those associated with ECM remodelling and immune response (Figure 4B). Among these, we identified a strong upregulation of MMP genes in injured *tert* mutant muscle compared to injured WT (Figure 4C, Table S1). Using qPCR and HCR, we confirmed elevated expression of *mmp9* and *mmp13* in *tert* mutant muscle following injury (Figure 4D,E).

Given that the regenerating muscle of *tert* mutants shows a strong inflammatory signature, which may perturb immune cell function, we examined macrophage responses to injury. In WT larvae, macrophages had left the injury site by 24 hpi, while in *tert* mutants, macrophages persisted at the site of injury (Figure 4F). Quantification of macrophages revealed significantly higher numbers in *tert* mutants at both 8 hpi and 24 hpi, indicative of a prolonged inflammatory response to injury in *tert* mutants and a failure of resolution (Figure 4G).

### 3.5 MMP9/13 Inhibitor Treatment Restores muSC Activation, Proliferation and Regeneration in *tert* Mutants

**FIGURE 5:**
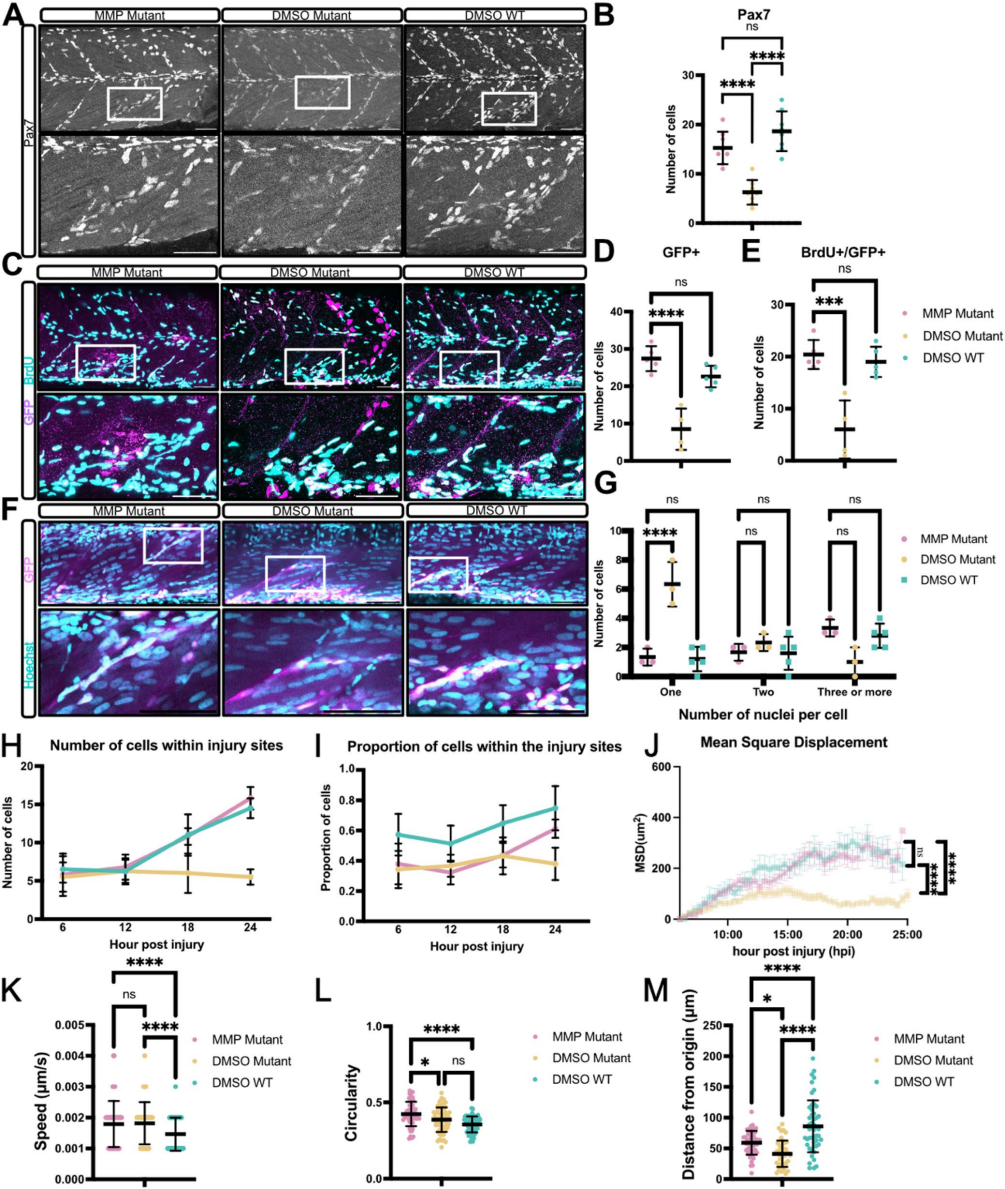
MMP9/13 inhibitor I treatment restores muSC activation, proliferation and migration in *tert* mutants. (A) Immunolabelling of Pax7+ muSCs in *tert* mutant and WT larvae treated with MMP9/13 inhibitor I. Number of animals used *n* = 10 (*tert* mutant + inhibitor), *n* = 8 (*tert* mutant + DMSO), *n* = 6 (WT + DMSO). (B) Quantification of muSCs expressing pax7a:egfp in muscle of injured *tert* mutant treated with MMP9/13 inhibitor compared to untreated *tert* mutant and WT. (C) Representative images of BrdU labelling (cyan) relative to muSCs expressing pax7a:eGFP (GFP, magenta). Number of animals used *n* = 12 (*tert* mutant + MMP inhibitor), *n* = 9 (*tert* mutant + DMSO), *n* = 7 (WT + DMSO). (D, E) Quantification of GFP positive (magenta) muSCs and muSCs expressing both BrdU (cyan) and GFP (magenta) in inhibitor treated *tert* mutant larvae and control groups. (F) Representative images of multinucleated myofibres following inhibitor treatment. (G) Quantification of multinucleated myofibres. Number of animals used *n* = 9 (*tert* mutant + MMP inhibitor), *n* = 6 (*tert* mutant + DMSO), *n* = 6 (WT + DMSO). Quantification of the number (H) and the proportion (I) of muSCs accumulating at the injury site over time in inhibitor treated *tert* mutant and control groups. (J) MSD analysis of muSC movement in treated *tert* mutant compared to untreated *tert* mutant and WT animals. (K–M) Quantification of muSC migration parameters, including mean cell speed (K), mean cell circularity (L) distance travelled from origin (M). Number of animals used *n* = 10 (*tert* mutant + MMP inhibitor), *n* = 8 (*tert* mutant + DMSO), *n* = 8 (WT + DMSO). Data are presented as mean ± SD and statistical significance was determined using an unpaired Student's *t*‐test (ns not significant, *p < 0.05; ***p < 0.001, ****p < 0.0001). Scale bars: 50 μm.

Metalloproteinases are major regulators of tissue remodelling and are expressed by macrophages during muscle injury (Ratnayake et al. 2021). Both *mmp9* and *mmp13* were significantly upregulated in injured *tert* mutant muscle compared to WT muscle, suggesting they may be important for the impaired muSC response. We therefore tested how MMP inhibition affects muSC responses to injury by treating *tert* mutant and WT larvae with MMP9/13 inhibitor I 24 h prior to injury, and subsequently after injury, then examined muSC responses. Relative to untreated *tert* mutants, the number of Pax7+ muSCs was significantly increased in *tert* mutants, reaching levels comparable to WT controls (Figure 5A,B). Similarly, proliferation of muSCs expressing pax7a:egfp was also significantly improved in inhibitor treated *tert* mutants compared to untreated *tert* mutants (Figure 5C–E). These results suggest that excessive MMP9/13 activity impairs muSC activation and proliferation, and its inhibition rescues these early regenerative responses.

To evaluate whether this improvement in stem cell function translates into improved muscle regeneration, we quantified multinucleated myofibres at 96 hpi. Inhibitor‐treated *tert* mutant larvae exhibited a significant increase in the number of multinucleated fibres, indicating enhanced fusion and improved regeneration (Figure 5F,G). Examination of myofibre morphology at 6 dpi using phalloidin revealed that the alignment of the newly regenerated fibres was similar to that seen in WT larvae (Figure S4A). Further characterisation of NMJ formation in regenerating muscle revealed an increased number of presynaptic and postsynaptic puncta at the injury site in inhibitor‐treated *tert* mutants relative to untreated control *tert* mutants. The number of co‐localised puncta in inhibitor‐treated *tert* mutants was comparable to that seen in regenerating WT larvae (Figure S5A,B).

These results demonstrate that excessive MMP9/13 activity contributes to the impaired regenerative phenotype in *tert* mutants, and inhibition of MMPs can effectively rescue muSC function and muscle regeneration.

### 3.6 Partial Restoration of muSC Migration by MMP9/13 Inhibition

In *tert* mutants, muSCs showed a reduced proliferative response to injury, which coincided with an altered migratory behaviour. To understand whether the rescue of muSC proliferation, differentiation, and fusion by MMP9/13 inhibition correlated with a rescue of cell behaviour, we tracked muSC migration from 6 hpi to 24 hpi in larvae treated with MMP9/13 inhibitor I and compared it to DMSO treated controls (Figure S6, Movie S3).

Quantitative analyses showed that MMP9/13 inhibition increased the number and proportion of muSCs reaching the injury site in *tert* mutants over time compared to untreated *tert* mutants (Figure 5H,I). Furthermore, analysis of migration trajectories revealed that muSCs displayed significantly greater displacement from their origin in inhibitor‐treated *tert* mutants compared to untreated *tert* mutants, indicative of partially restored migratory capacity (Figure 5J).

Despite observing a similar number of cells around the injury site (Figure 5H,I), as well as comparable MSD (Figure 5J) and mean cell speed (Figure 5K) between inhibitor‐treated *tert* mutants and WT controls, detailed analysis revealed that muSCs from inhibitor‐treated *tert* mutants still exhibited different migratory behaviours relative to WT animals. Specifically, there were proportionately fewer cells reaching the injury site (Figure 5I); they showed a shorter migratory distance from their origin (Figure 5M) and showed increased cell circularity compared to WT muSCs (Figure 5L). These observations indicate that although overall migratory behaviour and accumulation at the injury site were improved, certain aspects of the migratory pattern remained abnormal.

### 3.7 Macrophage Depletion Restores Muscle Stem Cell Activation and Proliferation in *tert* Mutants

To understand whether macrophage behaviour was affected by MMP9/13 inhibitor I treatment, we performed live imaging to visualise macrophage responses to injury. We observed fewer macrophages in the injured myotome of inhibitor‐treated *tert* mutants compared to untreated mutants (Figure S7). In wildtype zebrafish larvae, *mmp9*‐expressing macrophages are required for muSC proliferation (Ratnayake et al. 2021). We wondered whether elevated expression of *mmp9* in macrophages at the injury, coupled with a lack of resolution, could explain the impaired muSC response to injury in *tert* mutants. To better understand whether macrophage presence directly impacts muSC behaviour and proliferation in an ageing‐like context, we utilised the Metronidazole‐Nitroreductase (MTZ‐NTR) system to perform macrophage depletion under the control of the macrophage‐specific promoter *fms* (*csfr1a*) (Gray et al. 2011). Successful macrophage depletion was confirmed via fluorescence microscopy (Figure S8).

**FIGURE 6:**
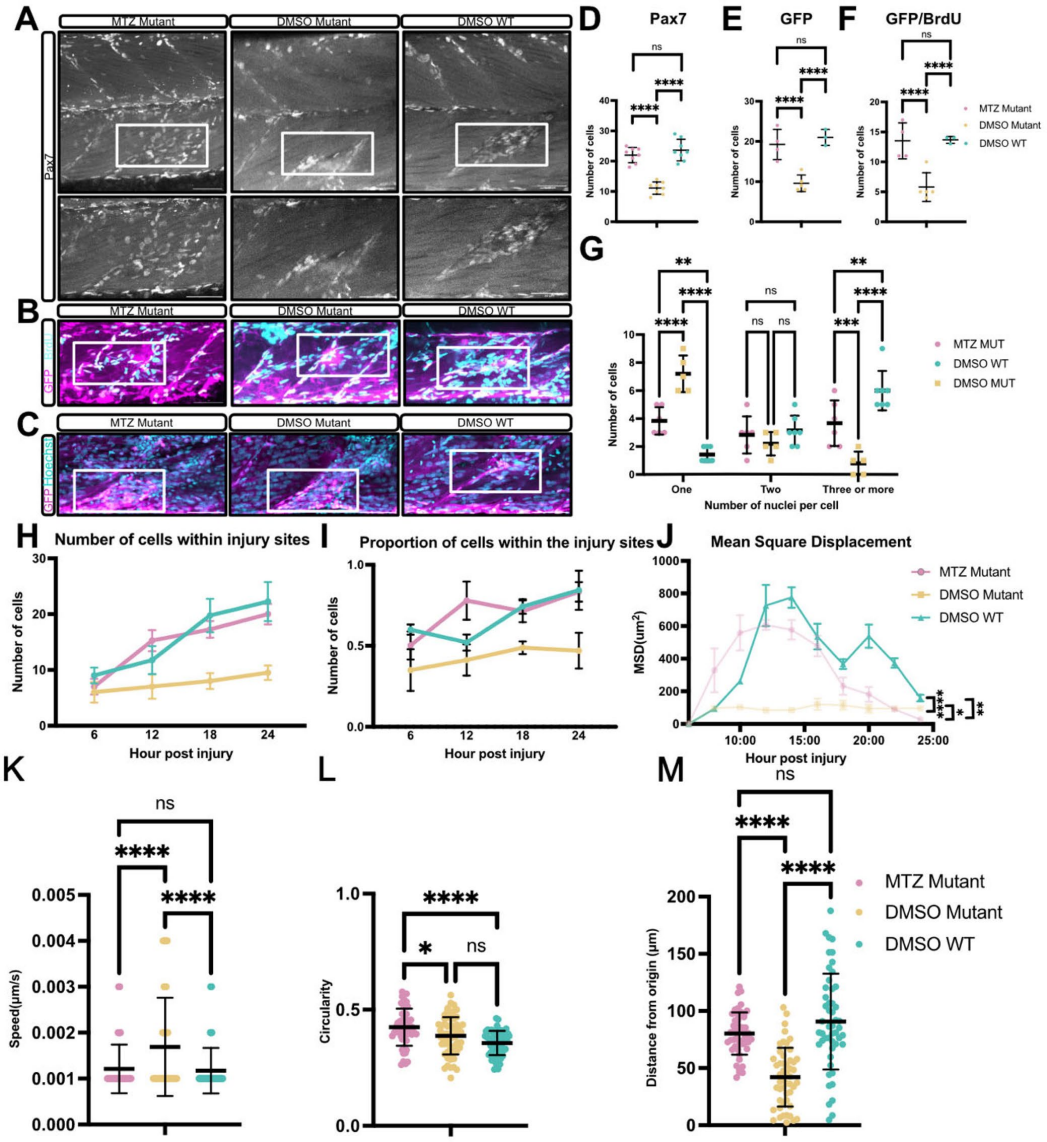
Macrophage depletion restores early muSC responses but does not rescue late stage muscle regeneration in *tert* mutant larvae. (A) Representative images of Pax7 staining in *tert* mutant and WT larvae with and without MTZ treatment. Number of animals used *n* = 8 (all conditions). (B) Representative images of BrdU (cyan) incorporation in larvae expressing pax7a:egfp (GFP, magenta). Number of animals used *n* = 4 (*tert* mutant + MTZ), *n* = 5 (*tert* mutant + DMSO), *n* = 3 (WT + DMSO). (C) Representative images of multinucleated myofibres following macrophage depletion. (D) Quantification of GFP positive muSCs in MTZ treated *tert* mutant larvae expressing pax7a:egfp and DMSO treated *tert* mutants. (E, F) Quantification of BrdU and BrdU/GFP double‐positive cells in MTZ treated *tert* mutant larvae expressing pax7a:egfp and DMSO treated *tert* mutants. (G) Quantification of multinucleated myofibres. Number of animals used *n* = 5 (*tert* mutant + MTZ), *n* = 4 (*tert* mutant + DMSO), *n* = 86 (WT + DMSO). Quantitative analyses show (H) the number of muSCs expressing pax7a:egfp at the injured muscle, (I) the proportion of cells within the injury sites relative to total muSC numbers within the myotome, (J) mean squared displacement (MSD), (K) mean cell speed, (L) cell circularity and (M) distances travelled from origin by muSCs. Number of animals used *n* = 6 (*tert* mutant + MTZ), *n* = 4 (*tert* mutant + DMSO) and *n* = 5 (WT + DMSO). Data are presented as mean ± SD and statistical significance was determined using an unpaired Student's *t*‐test (ns, not significant, *p < 0.05, **p < 0.01, ****p < 0.0001). Scale bars: 50 μm.

Following macrophage depletion, we observed a significant restoration in the number of Pax7 positive muSCs and an increase in BrdU incorporation by muSCs, indicating improved activation and proliferation of muSCs in *tert* mutants (Figure 6A,B,D–F). However, analysis at 6 dpi revealed persistent abnormalities in myofibre organisation in *tert* mutants following macrophage depletion (Figure S4B). To further evaluate the regeneration process, we quantified myofibres at 96 hpi. Inhibitor treated *tert* mutant larvae exhibited a slight increase in the number of multinucleated fibres, but not comparable with DMSO treated WT larvae (Figure 6C,G), consistent with persistent abnormalities observed. To determine whether re‐innervation had been rescued, NMJ formation in regenerating muscle was quantified. An increased number of co‐localised presynaptic and postsynaptic puncta were detected at the injury site in *tert* mutants lacking macrophages, but this was not comparable to WT larvae (Figure S5C,D). This suggests that removing macrophages did not fully rescue muscle regeneration in *tert* mutants.

Our findings therefore suggest that macrophage depletion partially restores early muSC responses to injury in *tert* mutants, but is insufficient to rescue normal regeneration.

### 3.8 Macrophage Depletion Alters muSC Migration Dynamics

To determine how macrophage depletion affected muSC migration dynamics during regeneration, we visualised muSCs expressing pax7a:egfp (Figure S9, Movie S4). We found that there was an earlier and more robust accumulation of muSCs at the injury site of *tert* mutants following macrophage ablation compared to untreated *tert* mutants (Figure 6I). This reflects an increased proportion of muSCs localising within the injury zone, suggesting recruitment of muSCs is faster in the absence of macrophages (Figure 6J).

Further analysis of migration characteristics showed that macrophage depletion partially restored several aspects of muSC migratory behaviour. MuSCs in treated *tert* mutants showed a significant increase in distance travelled from the point of origin (Figure 6N) and a reduction in cell circularity (Figure 6M), reflecting more efficient and directional movement. Mean cell speed was decreased in MTZ treated *tert* mutants relative to control mutants (Figure 6L), and although mean squared displacement (MSD) was significantly different compared to control mutants, it was also different from wildtype animals (Figure 6K).

These results indicate that macrophage depletion enhances initial muSC recruitment and partially rescues migratory behaviour in *tert* mutants. However, the altered dynamics of muSC recruitment in macrophage‐ablated *tert* mutant animals relative to wildtype animals, reveals macrophages are required for regulating normal behaviour of muSC responses to injury.

## 4 Discussion

Our study reveals that an ageing‐like environment is refractory to muscle regeneration in *tert* mutants, accompanied by impaired muSC activation and migratory dynamics. This is mediated by an inappropriate activity of metalloproteases secreted by macrophages, which inhibit muSC migration and generation of new myofibres. By deploying in vivo imaging, we demonstrate that the dynamics of muSC responses to injury can be linked to their functional capacity to repair muscle, linking cellular behaviour to regenerative outcomes. These findings underscore how chronic inflammatory and ECM changes to the muscle environment affect muSC function in vivo, providing new insight into the basis for impaired muscle regeneration in ageing.

Telomerase‐deficient zebrafish rapidly develop an ageing‐like phenotype, making them a powerful model to study how age‐related changes in the tissue environment impact regeneration. Telomere shortening in *tert* mutant triggers increased cellular senescence and chronic, systemic inflammation (Hernández‐Silva et al. 2025; Lex et al. 2020). While *tert* mutants do not fully recapitulate the entire spectrum of mammalian or naturally aged zebrafish muscle ageing, such as systemic metabolic decline or cumulative endocrine alterations, they offer a tractable model to interrogate specific ageing‐like stressors that emerge from telomere dysfunction. Our study focuses on how such stressors, including chronic inflammation and ECM remodelling, influence muscle stem cell behaviour during regeneration. Although there are developmental differences between larvae and adults, particularly in immune system maturation, core features of macrophage‐mediated repair—such as recruitment, debris clearance and pro‐regenerative polarisation—are preserved across life stages in zebrafish (Gurevich et al. 2019). Moreover, we observed macrophage accumulation and altered migratory patterns that mirror those seen in adult tissue injury models (Reuter et al. 2022).

In this study, we observed molecular changes in zebrafish *tert* mutant larvae that are similar to those described in ageing zebrafish muscle, involving upregulation of stress responsive gene expression and an exaggerated inflammatory response (Jia et al. 2025; Kijima et al. 2022; Wang et al. 2025). Similar to ageing in mice, muSCs in *tert* mutants have a delayed activation and proliferation response after injury, and often a fraction of the muSC pool becomes senescent (Kimmel et al. 2020). The *tert* mutant larvae recapitulate this age‐associated decline in function, evidenced by reduced muSC activation and proliferation after muscle damage. We also observe elevated expression of senescence markers *p16* and *p21* in *tert* mutant muscle, indicative of a senescent program, reminiscent of ageing mammalian muscle.

To further explore the relationship between larval and adult ageing phenotypes, we performed transcriptomic profiling of skeletal muscle from 18‐month‐old *tert* mutants. This revealed widespread gene expression changes consistent with muscle ageing, including disrupted muscle architecture, chronic inflammation, impaired regenerative signalling, ECM remodelling, DNA damage accumulation and mitochondrial dysfunction. Similar to *tert* mutant larvae, we noted genes involved in ECM remodelling (*mmp2*) and stress‐ or immune‐related genes (*c4b*, *eva1bb*, *vsig8a*, *trpm1b* and *btr24*) show an altered expression. Likewise, the activation of DNA damage response genes (*tp53*, *phlda3*, *mdm2* and *parp3*) in both larval stages and adult animals suggests telomere dysfunction at developmental stages can have long‐lasting impacts on organismal health (Bednarek et al. 2015). It is striking that F2 *tert* mutant larvae recapitulate key hallmarks of premature ageing, including early inflammatory and ECM remodelling, that are hallmarks of premature ageing in F1 mutants. This suggests tuning age‐associated modifiers in development can have a long‐lasting impact into adult life.

Our live imaging experiments have demonstrated how ageing‐associated changes in telomerase mutants can affect the interaction between muSCs and immune cells in a regeneration context. After injury or damage, macrophages infiltrate to phagocytose tissue, activate resident stem cells, and then mostly exit the injury site, allowing muSCs to proceed with regeneration. In *tert* mutants, we observed macrophages lingering at injury sites well beyond the acute phase and physically clustering around muSCs. This persistent macrophage presence correlated with muSCs retaining a rounded, non‐spreading state, failing to adopt the spreading morphology typically associated with effective migration and differentiation. Such rounded cell shapes have previously been described for migratory cells experiencing altered ECM conditions or physical constraints, as reported in studies of macrophages and neutrophils during ECM remodelling (De Pascalis and Etienne‐Manneville 2017; Travnickova et al. 2021, 2017). Similarly, ECM composition and stiffness have been shown to significantly influence cell shape and migration patterns, as changes in ECM stiffness guide cells toward injury sites and modulate their morphology and speed of movement (Jiang et al. 2020; Travnickova et al. 2021). Furthermore, inflammatory cells may actively restraining muSCs, either through direct cell–cell contact or continual exposure to inflammatory cytokines (De Pascalis and Etienne‐Manneville 2017). Recent intravital imaging studies in mice have begun to characterise muSC interactions with macrophages under disease conditions, demonstrating prolonged contacts between muSCs and macrophages, which negatively impact stem cell motility and regenerative outcomes (Sarde et al. 2025). In the study, muSC and macrophage interactions during muscular dystrophy are often characterised by macrophage‐derived factors restricting muSC migration and differentiation, leading to impaired regeneration. Our zebrafish analyses extend these observations by muSCs in an ageing‐like context, reinforcing the concept that an effective regenerative response requires an acute, transient inflammatory reaction, followed by timely resolution to allow the initiation of regeneration.

In *tert* mutant larvae, we observed elevated expression of *mmp2*, *mmp9* and *mmp13* after injury, similar to descriptions of elevated *MMP9* expression in the muscle of aged mice (Kanazawa et al. 2023). MMPs are well‐established regulators of muscle injury repair, orchestrating both immune cell infiltration and tissue remodelling. In young mice, inflammatory cells (such as neutrophils and macrophages) secrete MMPs like MMP9 early after muscle injury to degrade components of the basement membrane, allowing leukocytes to rapidly infiltrate damaged tissue (Chen and Li 2009; Kherif et al. 1999). This proteolytic clearance not only removes debris but also creates space for incoming cells and releases ECM‐bound signals that further recruit inflammatory cells. As regeneration progresses, other MMPs (e.g., MMP2 and MMP13) become active to remodel the ECM, which facilitates migrating muSCs to reach the injury and form new myofibres (Chen and Li 2009; Kanazawa et al. 2023; Ratnayake et al. 2021). Thus, in a young environment, MMPs play a pro‐regenerative role by balancing timely inflammation and creating a permissive scaffold for repair. Consistent with these roles, muscle injuries in MMP‐deficient models often show aberrant tissue repair. Inhibition or genetic ablation of MMP9 can delay macrophage infiltration and modify regeneration outcomes (Kherif et al. 1999), whereas excessive or prolonged MMP activity can be deleterious, as seen by chronic high MMP9 activity in dystrophic muscle exacerbating tissue damage and inflammation (Li et al. 2009). These observations suggest that precise regulation of MMP expression and activity is crucial for effective muscle regeneration.

Our findings point to two major extrinsic regulators of muSC behaviour in an ageing context: macrophage mediated inflammation and ECM remodelling. Both factors are known to influence muSC function in normal regeneration, and our analyses in telomerase deficient animals show how their dysregulation leads to defective outcomes. To determine how macrophage function contributes to this phenotype, we showed that ablation of macrophages in *tert* mutant partially restored muSC function and behaviour. However, we observed this resulted in altered cell dynamics with many more muSCs accumulating at the wound site more rapidly than observed in untreated *tert* mutant animals. This is presumably because inhibitory signals or physical blockage from macrophages are no longer present to prevent muSCs migrating into the damaged muscle area. Despite arriving at the injury site earlier in an absence of macrophages, the muSCs then pause, maintaining a rounded shape, prior to differentiation and fusion. This pattern of ‘gather early, integrate late’ suggests that persistent macrophages in an ageing‐like environment interfere with the initial phase of muSC recruitment to injury.

Conversely, when MMP activity is inhibited in *tert* mutants, muSCs show a more directional and faster migration, which is more similar to WT animals as previously described (Brondolin et al. 2023). This suggests that MMP9/13 inhibition has restored sufficient cues from the surrounding environment to enable a more normal muSC movement. However, even though muSC arrival timing improved, the efficiency of regeneration remained low, evidenced by a smaller proportion of muSCs at the injury site in *tert* mutants compared to WT. Differences in the type of behavioural changes to muSCs in *tert* mutants in response to macrophage removal or MMP inhibition suggest these perturbations are affecting different cell responses. Macrophage depletion increased the speed of muSC recruitment to the injury, whereas MMP inhibition rescued normal migratory behaviour.

Based on these observations, we propose that macrophages and ECM components play complementary but distinct roles in regulating muSC behaviour in ageing. Persistent macrophages maintain an inflammatory environment and may physically hinder muSCs, thereby delaying their initial activation and migration to injuries. On the other hand, altered ECM modelling creates a suboptimal migratory landscape for muSCs, affecting how efficiently they can travel. Effective muscle regeneration requires both timely inflammation resolution and an appropriate remodelling of the ECM; ageing skews both processes, and each must be addressed to rejuvenate regenerative capacity.

These findings advance our understanding of muscle ageing by demonstrating that a combination of niche‐extrinsic ageing changes leads to regenerative failure. Using in vivo imaging, we reveal how muSC function is driven by a dynamic response to macrophage‐dependent cues, which are impaired in ageing due to elevated MMP activity. We also highlight that the zebrafish *tert* mutant recapitulates many of the changes observed in ageing muscle and offers a powerful system for identifying ageing‐associated factors driving the aberrant behaviour of distinct cell populations in tissue homeostasis and repair.

## Author Contributions

Carlene Dyer and Yue Yuan were involved in investigation and formal analysis. Yue Yuan was involved in data curation, visualisation and writing – original draft. Robert Knight was involved in funding acquisition, supervision and writing – review and editing. Yue Yuan and Robert Knight were involved in methodology and conceptualisation.

## Conflicts of Interest

The authors declare no conflicts of interest.

## Supporting information


**Movie S1:** Representative live imaging of WT larvae expressing the pax7a:egfp transgene. Scale bar: 50 μm.

**Movie S2:** Representative live imaging of *tert* mutant larvae expressing the pax7a:egfp transgene. Scale bar: 50 μm.

**Movie S3:** Representative live imaging of MMP9/13 inhibitor treated *tert* mutant larvae expressing the pax7a:egfp transgene. Scale bar: 50 μm.

**Movie S4:** Representative live imaging of macrophage ablation *tert* mutant larvae expressing the pax7a:egfp transgene. Scale bar: 50 μm.

**Table S1:** Differentially expressed genes showing significant differences between conditions.

**Table S2:** Comparison of MSD for *tert* mutant versus wildtype.
**Table S3:** Comparison of MSD for MMP9/13 inhibitor treated *tert* mutant versus wildtype.
**Table S4:** Comparison of MSD for MTZ treated *tert* mutant versus wildtype.
**Figure S1:** muSC proliferation is reduced in *tert* mutant zebrafish under homeostatic conditions.
**Figure S2:** Transcriptome profiling of 18 month *tert* mutant and heterozygous animals.
**Figure S3:** No significant difference in Acridine Orange positive cells in injured *tert* mutant and WT zebrafish muscle.
**Figure S4:** Muscle fibres alignment in MMP 9/13 inhibitor I treated and MTZ treated *tert* mutant following injury.
**Figure S5:** Characterision of of NMJs at 6dpi following MMP9/13 inhibitor treatment or MTZ ablation of macrophages.
**Figure S6:** MMP9/13 inhibition enhances muSC migration to injured muscle of *tert* mutants.
**Figure S7:** MMP9/13 inhibition reduces general macrophage accumulation at the injury site in *tert* mutant larvae.
**Figure S8:** Evaluation of macrophage depletion in MTZ treated larvae.
**Figure S9:** macrophage ablation rescues muSC migration to the injury site in *tert* mutants.

## Data Availability

The RNA sequencing data that support the findings of this study are openly available in Array Express at https://www.ebi.ac.uk/biostudies/arrayexpress with accession number E‐MTAB‐15577. Other data are available in Appendix S1. Raw image files are available from the authors on request.
