# Supplementary material for: Muscle Regeneration Can Be Rescued in a Telomerase Deficient Zebrafish Model of Ageing by MMP Inhibition
Source: Aging Cell. 2025 Sep 25;24(11):e70238. doi: 10.1111/acel.70238 (PMC12608090; doi:10.1111/acel.70238)
Supplement: Supplementary file 5 — Table S1: Differentially expressed genes showing significant differences between conditions. [file ACEL-24-e70238-s001.zip › TableS1_caption.docx]

**Supplementary Table 1: Differentially expressed genes showing significant differences between conditions.**

Normalised gene expression counts (baseMean) for gene identities derived from the Zebrafish reference genome build 11 (Danio_rerio.GRCz11) were compared between conditions to generate values of log2fold changes with standard error (lfcSE), a z-statistic and corresponding p value, with adjusted p-values (padj) for multiple testing (Benjamini-Hochberg). Conditions tested are shown on the corresponding worksheet and are 1) muscle from uninjured 5 day old wildtype (WT) and tert mutants (MUT), 2) muscle from 24 hour post injury 5 day old wildtype (WT) and tert mutants (MUT), 3) muscle from uninjured 18 month old tert heterozygote (Hets) and tert mutant (MUT) animals.
