# Supplementary material for: Muscle Regeneration Can Be Rescued in a Telomerase Deficient Zebrafish Model of Ageing by MMP Inhibition
Source: Aging Cell. 2025 Sep 25;24(11):e70238. doi: 10.1111/acel.70238 (PMC12608090; doi:10.1111/acel.70238)
Supplement: Supplementary file 6 — Table S2: Comparison of MSD for tert mutant versus wildtype. Table S3: Comparison of MSD for MMP9/13 inhibitor treated tert mutant versus wildtype. Table S4: Comparison of MSD for MTZ treated tert mutant versus wildtype. Figure S1: muSC proliferation is reduced in tert mutant zebrafish under homeostatic conditions. Figure S2: Transcriptome profiling of 18 month tert mutant and heterozygous animals. Figure S3: No significant difference in Acridine Orange positive cells in injured tert mutant and WT zebrafish muscle. Figure S4: Muscle fibres alignment in MMP 9/13 inhibitor I treated and MTZ treated tert mutant following injury. Figure S5: Characterision of of NMJs at 6dpi following MMP9/13 inhibitor treatment or MTZ ablation of macrophages. Figure S6: MMP9/13 inhibition enhances muSC migration to injured muscle of tert mutants. Figure S7: MMP9/13 inhibition reduces general macrophage accumulation at the injury site in tert mutant larvae. Figure S8: Evaluation of macrophage depletion in MTZ treated larvae. Figure S9: macrophage ablation rescues muSC migration to the injury site in tert mutants. [file ACEL-24-e70238-s002.docx]

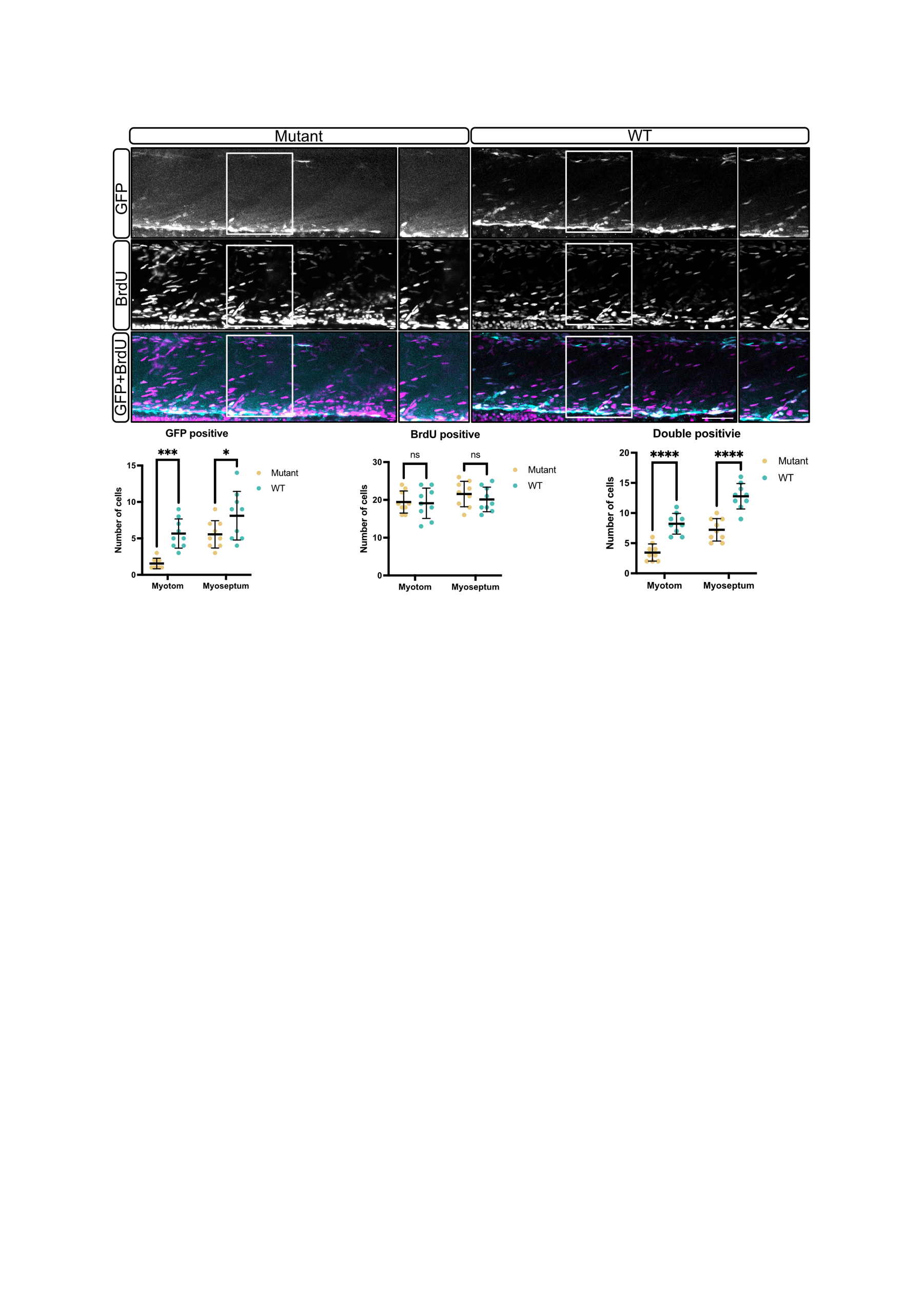


**Supplementary Figure 1: muSC proliferation is reduced in *tert* mutant zebrafish under homeostatic conditions**

**Representative images and quantification of muscle in 9 dpf uninjured *tert* mutant and WT larvae expressing pax7a:egfp following 5 days of BrdU exposure. Top panels show GFP (magenta, positive cells), middle panels show BrdU labelling (cyan; proliferating cells), and bottom panels display merged GFP/BrdU image. Quantification of GFP positive, BrdU positive, and GFP/BrdU double-positive cells was performed in both the myotome and myoseptum. Number of animals used n = 9 (all conditions). Data shown as mean ± SD and statistical testing performed using an unpaired Student’s t-test (**ns not significant, * p < 0.05, *** p < 0.001, **** p < 0.0001) **Scale bars: 50 μm.**


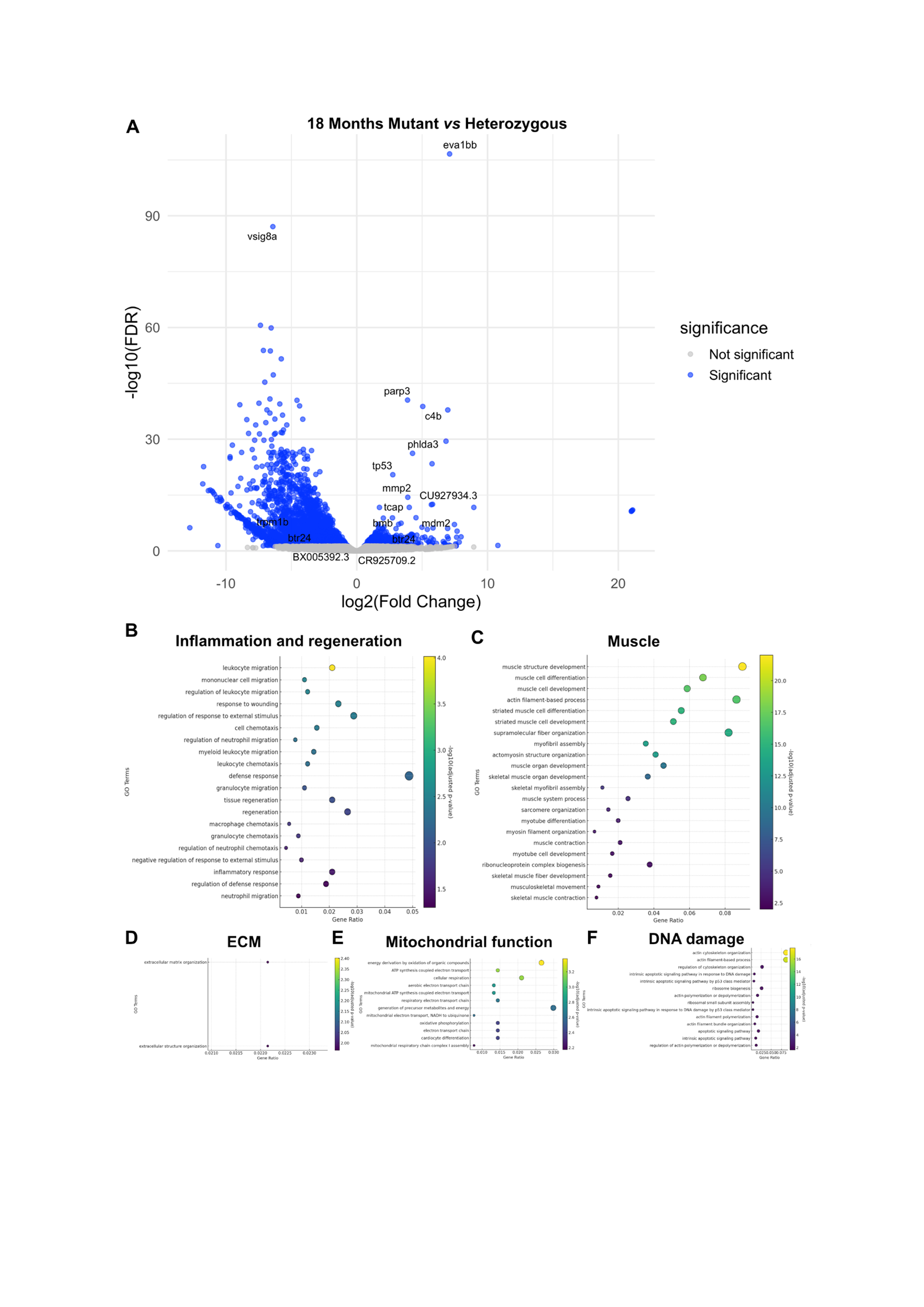


**Supplementary Figure 2: Transcriptome profiling of 18 month *tert* mutant and heterozygous animals.**

**(A) Volcano plot showing differentially expressed genes (DEGs) in skeletal muscle from 18-month-old *tert* mutant fish compared to heterozygous siblings. Significantly up- or downregulated genes (FDR<0.05) are highlighted in blue. (B-F) GO enrichment analysis of different categories in 18-month-old *tert* mutants.**


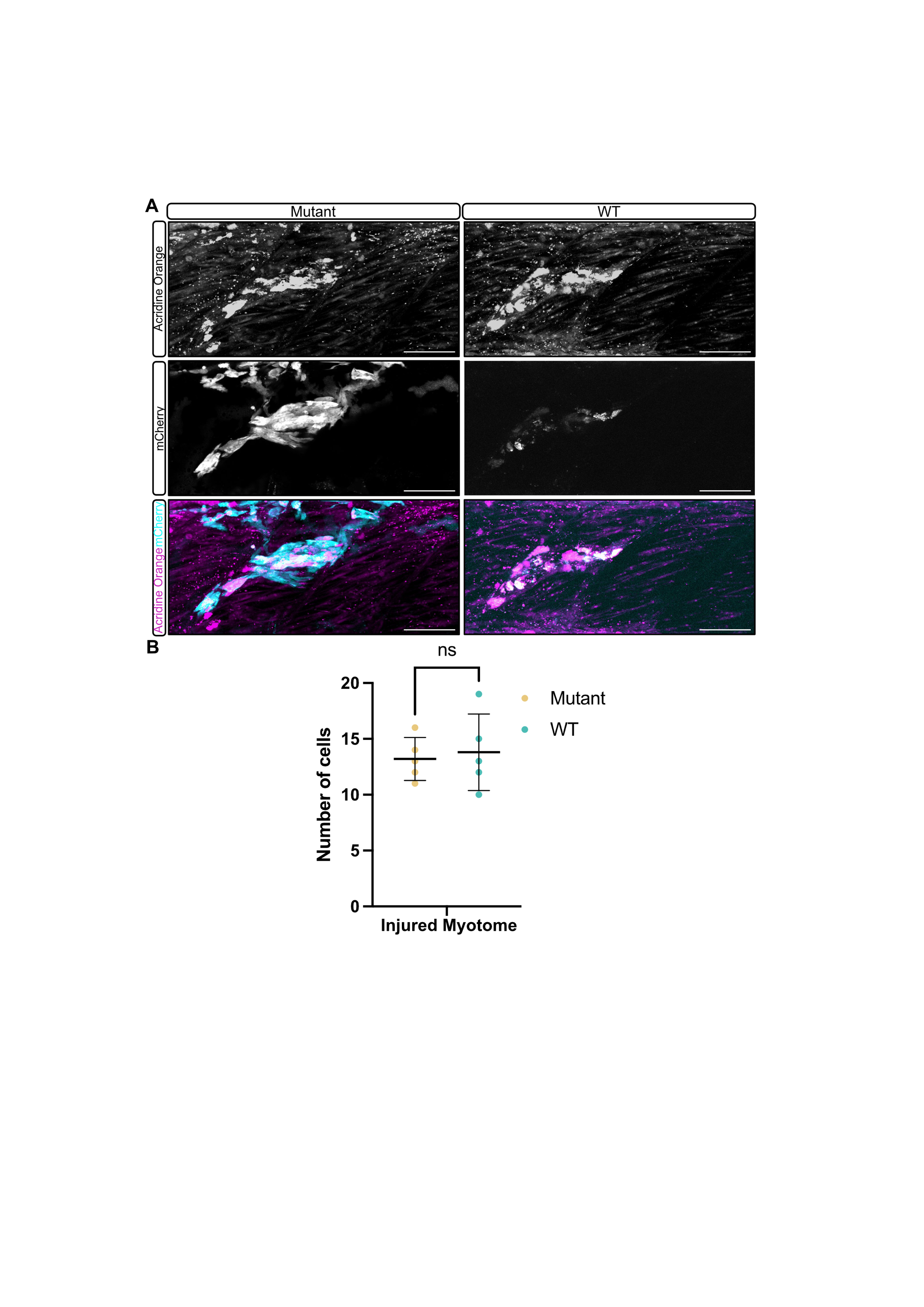


**Supplementary Figure 3: No significant difference in Acridine Orange positive cells in injured *tert* mutant and WT zebrafish muscle**

**(A) Representative images of injured myotomes in 5 dpf *tert* mutant and WT zebrafish larvae expressing fms:mCherry stained with Acridine Orange. (B) Quantification of Acridine Orange positive cells in the injured myotome. Number of animals used n = 5 (all conditions). Data shown as mean ± SD and statistical testing performed using an unpaired Student’s t-test (**ns not significant). **Scale bars: 50 μm.**


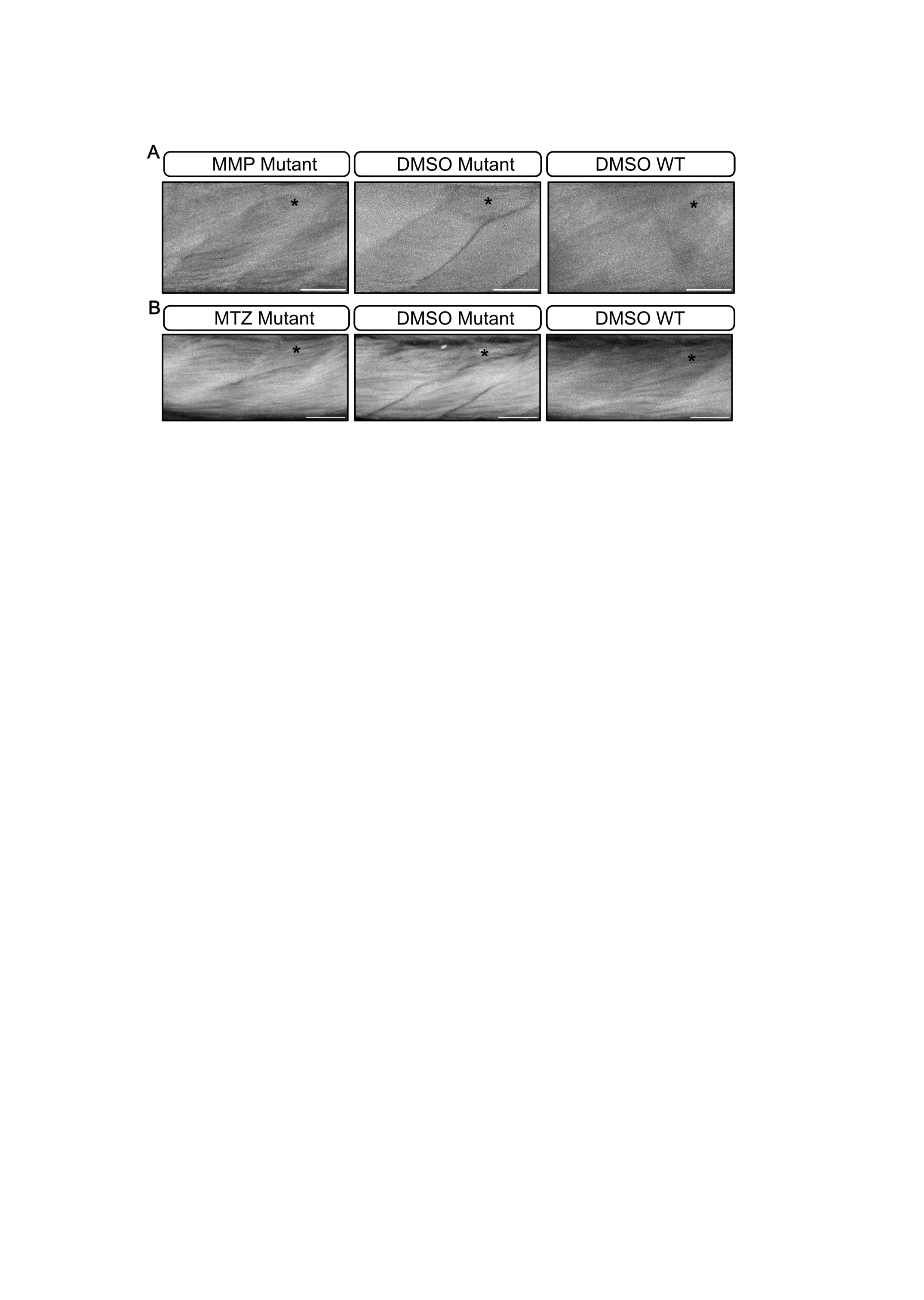


**Supplementary Figure 4. Muscle fibres alignment in MMP 9/13 inhibitor I treated and MTZ treated *tert* mutant following injury**

**(A–B) Representative phalloidin labelling of muscle at 6 dpi, showing myofibres morphology in larvae. (A) *tert* mutants treated with MMP9/13 Inhibitor I or DMSO, or WT treated with DMSO. (B) *tert* mutants treated with MTZ, *tert* mutant treated with DMSO and WT treated with DMSO. Asterisks mark the injury site. Scale bars: 50 μm.**


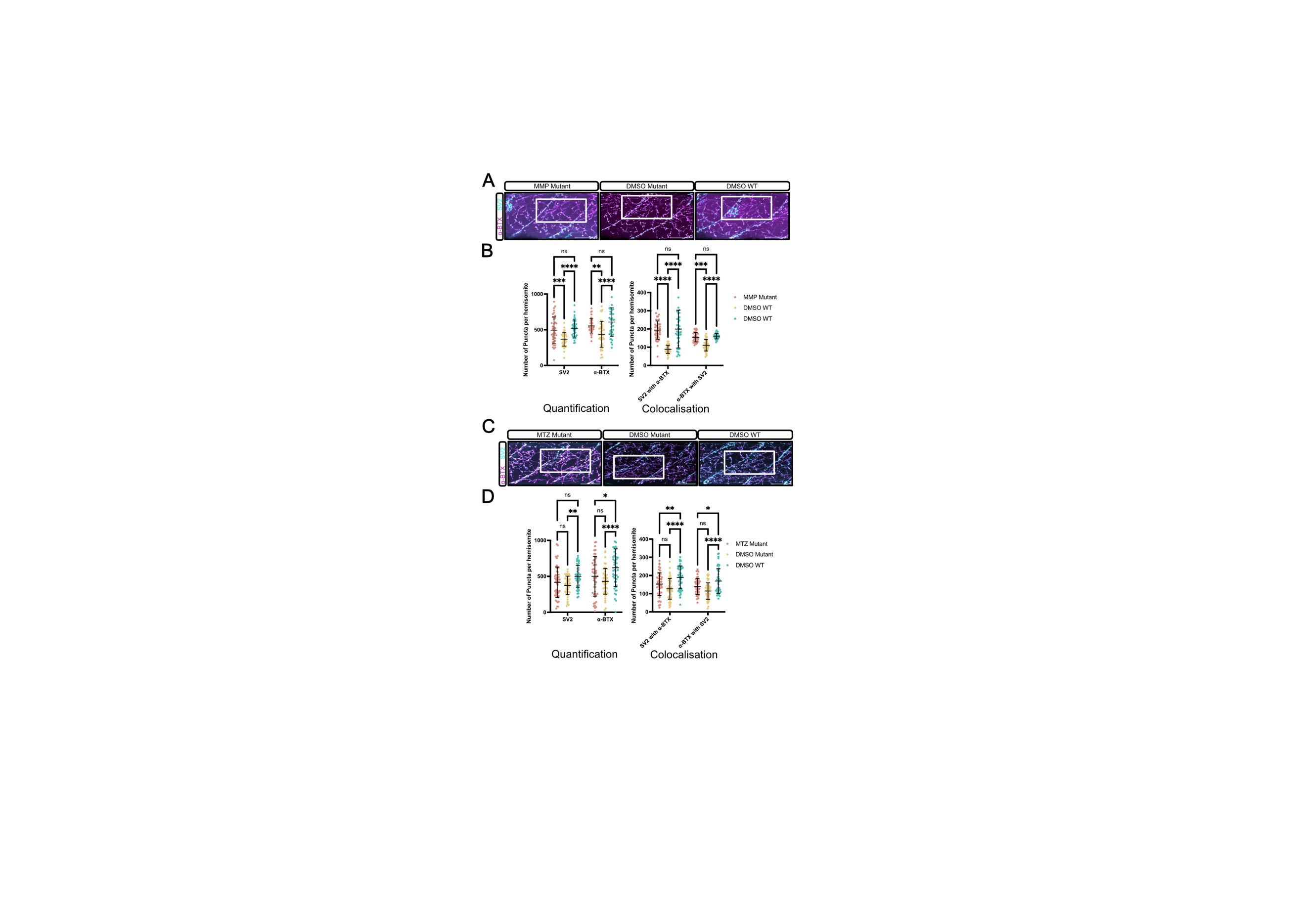


**Supplementary Figure 5: Characterision of NMJs at 6dpi following MMP9/13 inhibitor treatment or MTZ ablation of macrophages.**

**(A) and (C) Representative images of NMJs at 6dpi using** α-bungarotoxin (α-BTX; magenta) to detect AChR and SV2 (cyan) immunostaining to detect pre-synaptic vesicles after MMP9/13 inhibitor treatment (A) and macrophage depletion (C). **(B)** Quantification of SV2 or α-BTX puncta, and puncta showing colocalisation in muscle in larvae following macrophage depletion. Number of animals used MMP9/13 inhibitor treatment n=5 (for all conditions), n = 8 (*tert* mutant + MTZ), n = 6 (*tert* mutant + DMSO), n = 7 (WT + DMSO). Data are presented as mean ± SD and statistical significance was determined using an unpaired Student’s t-test (ns not significant, * p < 0.05, ** p < 0.01, **** p < 0.0001). Scale bars: 50 μm.


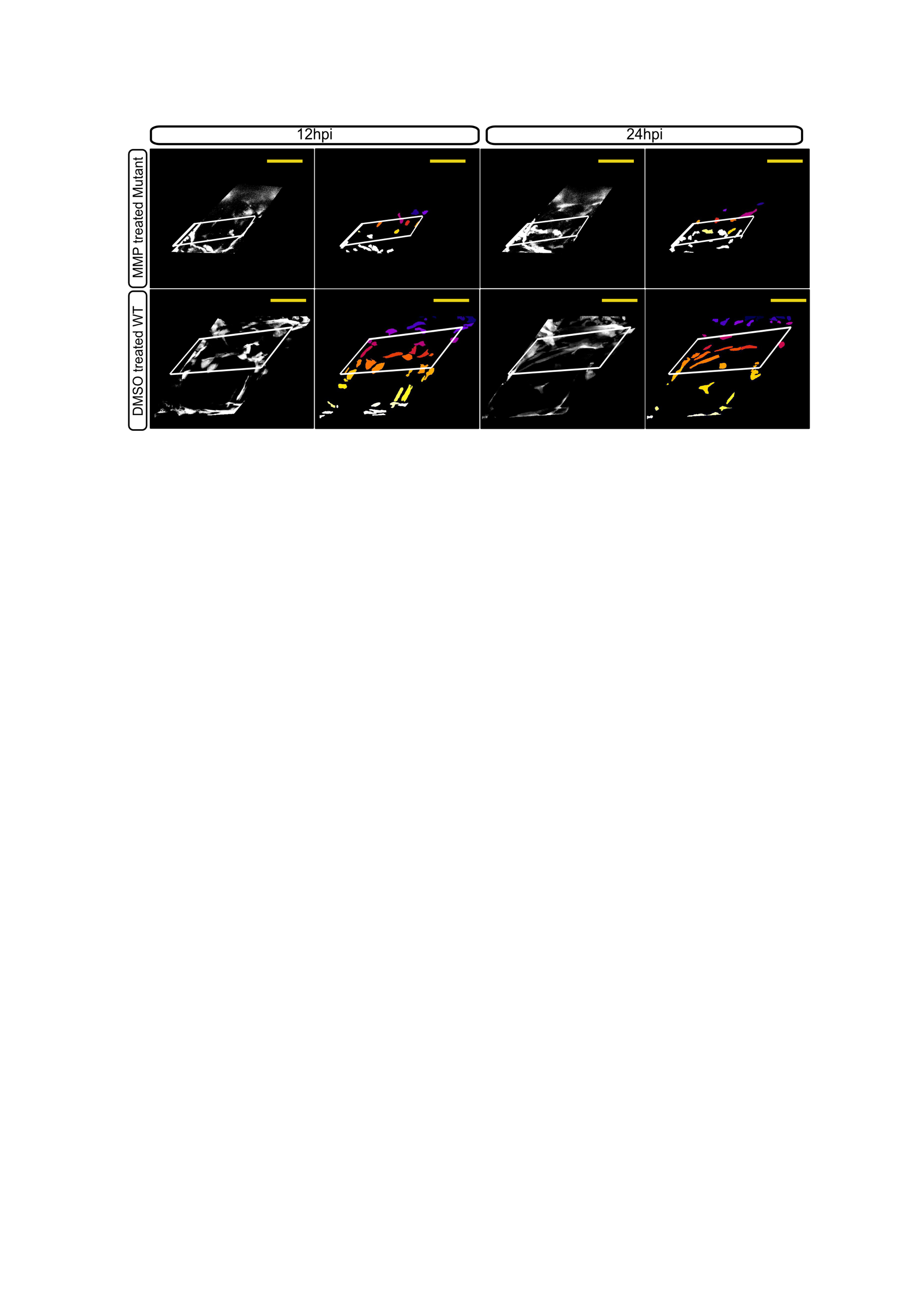


**Supplementary Figure 6: MMP9/13 inhibition enhances muSC migration to injured muscle of *tert* mutants**

**Representative images from time-lapsed recordings and corresponding segmented images of GFP positive muSCs in injured muscle of larvae expressing pax7a:egfp at 12 and 24 hpi. 5 dpf *tert* mutants were treated with MMP9/13 Inhibitor I 24 hours prior to injury and WT larvae were treated with DMSO. Scale bar 50 μm.**


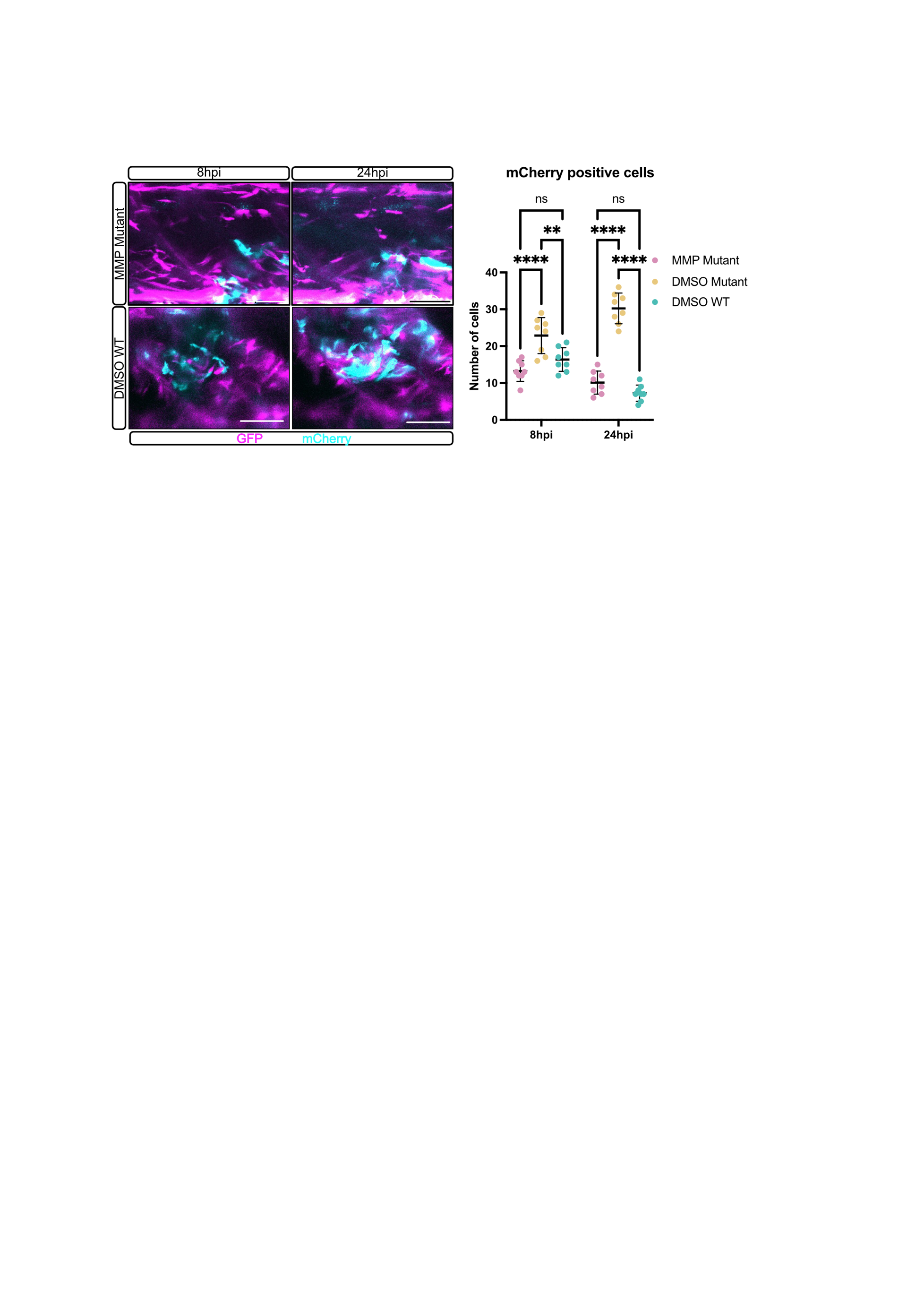


Macrophage

**Supplementary Figure 7: MMP9/13 inhibition reduces general macrophage accumulation at the injury site in *tert* mutant larvae.**

**Representative live images (left) and quantification (right) of macrophages in injured muscle of *tert* mutant and WT zebrafish larvae treated with MMP9/13 inhibitor I or DMSO (control) at 8 hpi and 24 hpi. Images of muSCs expressing pax7a:egfp (magenta) and macrophages expressing fms:mCherry (cyan) were captured by time-lapsed imaging. Number of animals used n = 10 (*tert* mutants treated with MMP Inhibitor I), n= 8 (*tert* mutants treated with DMSO), n = 8 (WT treated with DMSO). Data shown as mean ± SD and statistical testing performed using an unpaired Student’s t-test (**ns not significant, ** p < 0.01, *** p < 0.001, **** p < 0.0001). **Scale bars: 50 μm.**


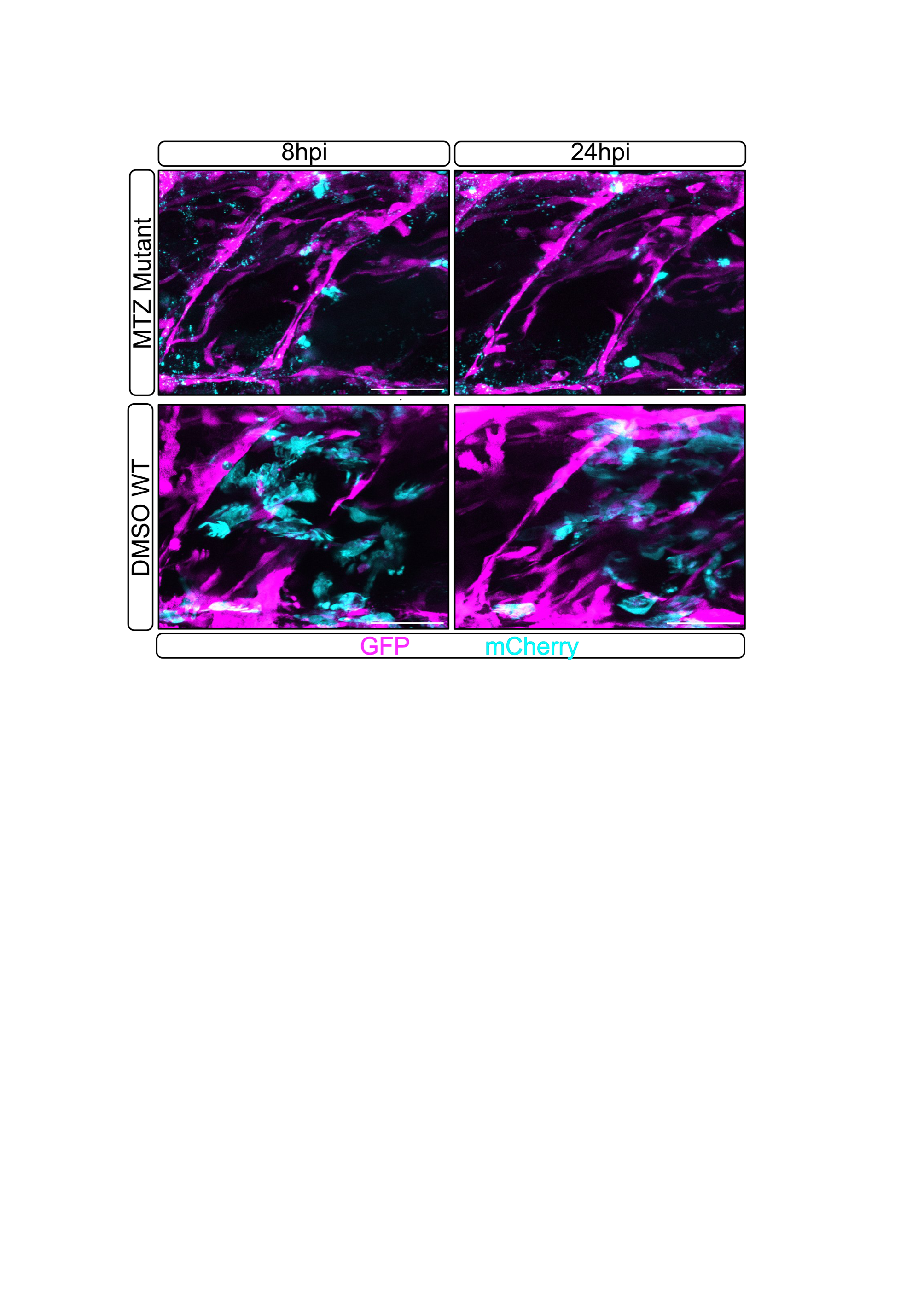


**Supplementary Figure 8: Evaluation of macrophage depletion in MTZ treated larvae.**

Representative images of *tert* mutant and WT larvae expressing pax7a:egfp (GFP, magenta) and fms:mCherry (mCherry, Cyan) transgenes at 8 and 24hpi after treatment with MTZ (*tert* mutant) or DMSO (WT). Scale bars: 50 μm.

**
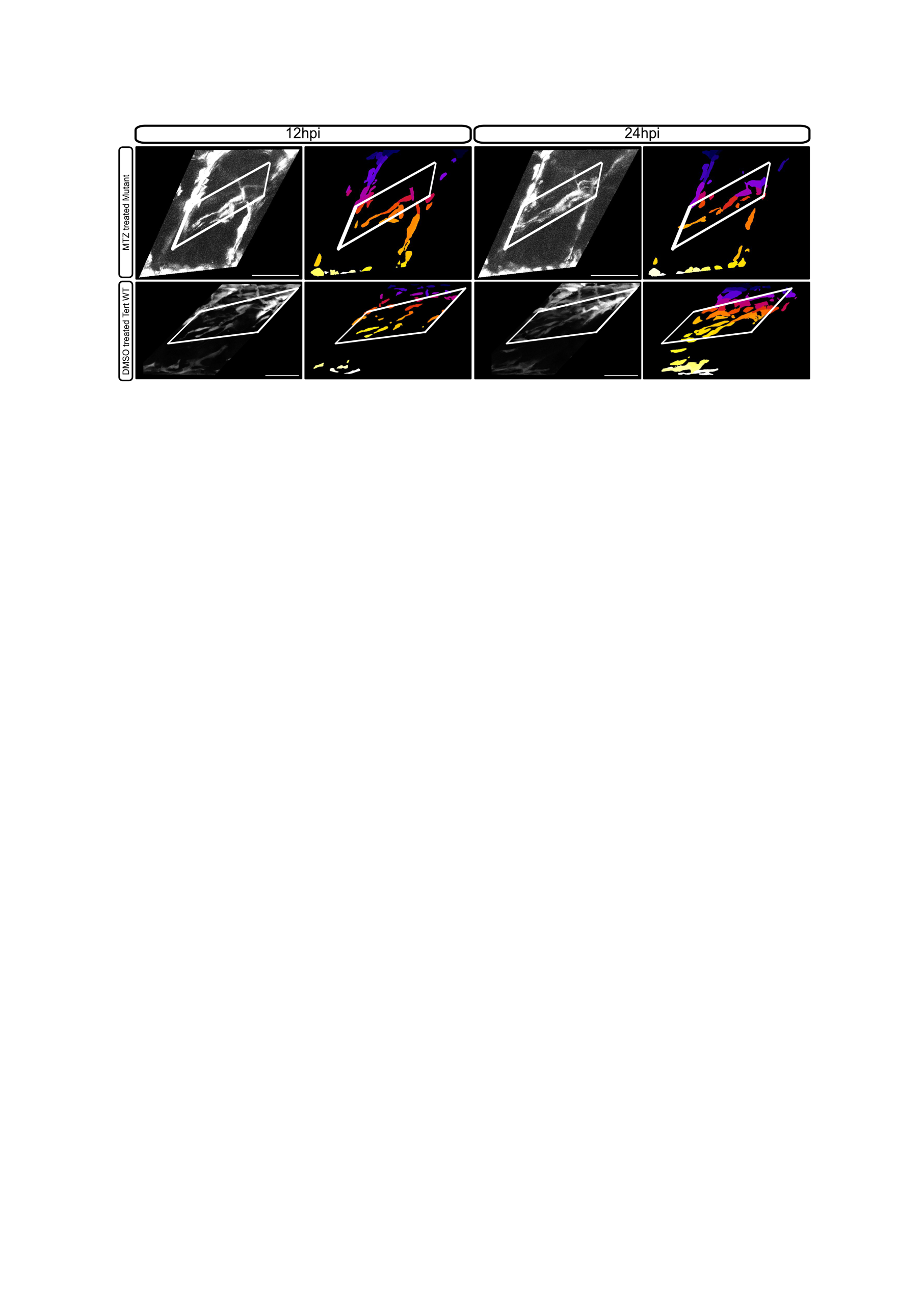
**

**Supplementary Figure 9: macrophage ablation rescues muSC migration to the injury site in *tert* mutants**

**Representative images from time-lapsed recordings and corresponding segmented images of GFP positive muSCs in injured muscle of larvae expressing pax7a:egfp at 12 and 24 hpi. 5 dpf *tert* mutants expressing fms:mCherry were treated with MTZ 24 hours prior to injury and WT larvae were treated with DMSO. Scale bar** 50 μm.

**List of supplementary Movies**

**Supplementary Movie 1: Representative live imaging of WT larvae expressing the pax7a:egfp transgene. Scale bar: 50 μm**

**Supplementary Movie 2: Representative live imaging of *tert* mutant larvae expressing the pax7a:egfp transgene. Scale bar: 50 μm**

**Supplementary Movie 3: Representative live imaging of MMP9/13 inhibitor treated *tert* mutant larvae expressing the pax7a:egfp transgene. Scale bar: 50 μm**

**Supplementary Movie 4: Representative live imaging of macrophage ablation *tert* mutant larvae expressing the pax7a:egfp transgene. Scale bar: 50 μm**

**Supplementary Table 2: Comparison of MSD for *tert* mutant vs wildtype**

Mixed effects multiple linear regression models of muSC movement in response to injury, application of genotype and time. 95% confidence intervals are shown for the linear coefficient of fit.

**Mixed-effects Linear Regression Summary**: **test for effect of genotype on MSD**

=======================================

Number of observations: 510

Number of groups: 17 (subject_id)

Fixed Effects

**Compared with WT**

+---------------+---------+-------------+---------+------------+----------------+----------------+

| Variable | Coef. | Std. Err. | z | P>|z| | 95% CI Lower | 95% CI Upper |

+===============+=========+=============+=========+============+================+================+

| genotype_mut | 0.1782 | 0.0365 | 4.8800 | 0.0000**** | 0.1069 | 0.2495 |

+---------------+---------+-------------+---------+------------+----------------+----------------+

| time | 0.0251 | 0.0034 | 7.3800 | 0.0000**** | 0.0186 | 0.0316 |

+---------------+---------+-------------+---------+------------+----------------+----------------+

| genotype#time | -0.0097 | 0.0022 | -4.4100 | 0.0000**** | -0.0140 | -0.0054 |

+---------------+---------+-------------+---------+------------+----------------+----------------+

| _cons | 0.6123 | 0.0571 | 10.7200 | 0.0000**** | 0.5004 | 0.7242 |

+---------------+---------+-------------+---------+------------+----------------+----------------+

Random Effects

+-----------------+------------+-------------+----------------+----------------+

| Random Effect | Estimate | Std. Err. | 95% CI Lower | 95% CI Upper |

+=================+============+=============+================+================+

| var(_cons) | 0.0354 | 0.0091 | 0.0202 | 0.0621 |

+-----------------+------------+-------------+----------------+----------------+

| var(Residual) | 0.0817 | 0.0074 | 0.0680 | 0.0982 |

+-----------------+------------+-------------+----------------+----------------+

Model Fit

----------

Log likelihood = -782.936

Wald chi2(3) = 142.71

Prob > chi2 = 0.0000

LR test vs. linear regression: chibar2(01) = 64.27, Prob >= chibar2 = 0.0000

**Supplementary Table 3: Comparison of MSD for MMP9/13 inhibitor treated *tert* mutant vs wildtype**

Mixed effects multiple linear regression models of muSC movement in response to injury, application of MMP9/13 inhibitor treatment and genotype. 95% confidence intervals are shown for the linear coefficient of fit.

**Mixed-effects Linear Regression Summary: test for effect of MMP9/13 inhibitor treatment on MSD**

**===========================================================**

**Number of observations: 676**

**Number of groups: 13 (subject_id)**

**Fixed Effects**

**Compared with DMSO_WT**

**+------------+---------+-------------+---------+------------+----------------+----------------+**

**| Variable | Coef. | Std. Err. | z | P>|z| | 95% CI Lower | 95% CI Upper |**

**+============+=========+=============+=========+============+================+================+**

**| DMSO_MUT | -0.1923 | 0.0371 | -5.1800 | 0.0000**** | -0.2649 | -0.1197 |**

**+------------+---------+-------------+---------+------------+----------------+----------------+**

**| MMP_MUT | 0.0064 | 0.0393 | 0.1600 | 0.8732 | -0.0703 | 0.0831 |**

**+------------+---------+-------------+---------+------------+----------------+----------------+**

**| time | 0.0225 | 0.0026 | 8.6500 | 0.0000**** | 0.0174 | 0.0276 |**

**+------------+---------+-------------+---------+------------+----------------+----------------+**

**| _cons | 0.5793 | 0.0491 | 11.8000 | 0.0000**** | 0.4831 | 0.6755 |**

**+------------+---------+-------------+---------+------------+----------------+----------------+**

**Random Effects**

**+-----------------+------------+-------------+----------------+----------------+**

**| Random Effect | Estimate | Std. Err. | 95% CI Lower | 95% CI Upper |**

**+=================+============+=============+================+================+**

**| var(_cons) | 0.0371 | 0.0092 | 0.0218 | 0.0613 |**

**+-----------------+------------+-------------+----------------+----------------+**

**| var(Residual) | 0.0804 | 0.0073 | 0.0667 | 0.0968 |**

**+-----------------+------------+-------------+----------------+----------------+**

**Model Fit**

**----------**

**Log likelihood = -753.841**

**Wald chi2(3) = 140.82**

**Prob > chi2 = 0.0000**

**LR test vs. linear regression: chibar2(01) = 70.91, Prob >= chibar2 = 0.0000**

**Contrasts**

**---------**

**MMP_MUT vs DMSO_MUT:**

**Difference = 0.1987**

**z = 5.01**

**p = 0.0000******

**95% CI: [0.1205, 0.2769]**

**Supplementary Table 4: Comparison of MSD for MTZ treated *tert* mutant vs wildtype**

Mixed effects multiple linear regression models of muSC movement in response to injury, application of macrophage ablation and genotype. 95% confidence intervals are shown for the linear coefficient of fit.

**Mixed-effects Linear Regression Summary: test for effect of macrophage ablation on MSD**

=======================================================================

Number of observations: 195

Number of groups: 15 (subject_id)

Fixed Effects

**Compared with DMSO_WT**

+------------+---------+-------------+---------+------------+----------------+----------------+

| Variable | Coef. | Std. Err. | z | P>|z| | 95% CI Lower | 95% CI Upper |

+============+=========+=============+=========+============+================+================+

| DMSO_MUT | -0.1862 | 0.0327 | -5.7000 | 0.0000**** | -0.2539 | -0.1185 |

+------------+---------+-------------+---------+------------+----------------+----------------+

| MTZ_MUT | 0.1327 | 0.0542 | 2.4500 | 0.0143** | 0.0264 | 0.2390 |

+------------+---------+-------------+---------+------------+----------------+----------------+

| time | 0.0229 | 0.0029 | 7.9000 | 0.0000**** | 0.0173 | 0.0285 |

+------------+---------+-------------+---------+------------+----------------+----------------+

| _cons | 0.5612 | 0.0507 | 11.0700 | 0.0000**** | 0.4619 | 0.6605 |

+------------+---------+-------------+---------+------------+----------------+----------------+

Random Effects

+-----------------+------------+-------------+----------------+----------------+

| Random Effect | Estimate | Std. Err. | 95% CI Lower | 95% CI Upper |

+=================+============+=============+================+================+

| var(_cons) | 0.0346 | 0.0091 | 0.0197 | 0.0603 |

+-----------------+------------+-------------+----------------+----------------+

| var(Residual) | 0.0789 | 0.0070 | 0.0654 | 0.0951 |

+-----------------+------------+-------------+----------------+----------------+

Model Fit

----------

Log likelihood = -749.672

Wald chi2(3) = 132.87

Prob > chi2 = 0.0000

LR test vs. linear regression: chibar2(01) = 67.48, Prob >= chibar2 = 0.0000

Contrasts

---------

MTZ_MUT vs DMSO_MUT:

Difference = 0.3189

z = 2.00

p = 0.0450*

95% CI: [0.0523, 0.4967]

**Supplementary Materials and Methods**

**Primers for qRT-PCR**

| Target name | Label | Reference |
| --- | --- | --- |
| il1b | FAM | (Progatzky et al., 2019) |
| cxcl18b | FAM | (Ramel et al., 2021) |
| mmp13a | FAM | (Rochon et al., 2020) |
| mmp9 | FAM | (Ramel et al., 2021) |

**References**

Progatzky, F., Jha, A., Wane, M., Thwaites, R. S., Makris, S., Shattock, R. J., Johansson, C., Openshaw, P. J., Bugeon, L., Hansel, T. T., & Dallman, M. J. (2019). Induction of innate cytokine responses by respiratory mucosal challenge with R848 in zebrafish, mice, and humans. *Journal of Allergy and Clinical Immunology*, *144*(1), 342-345.e7. https://doi.org/10.1016/j.jaci.2019.04.003

Ramel, M.-C., Progatzky, F., Rydlova, A., Wane, M., Schymeinsky, J., Williams, C., Jung, B., Lamb, J., Thomas, M. J., Bugeon, L., & Dallman, M. J. (2021). *Dynamics of repair and regeneration of adult zebrafish respiratory gill tissue after cryoinjury* (p. 2021.05.27.445469). bioRxiv. https://doi.org/10.1101/2021.05.27.445469

Rochon, E. R., Missinato, M. A., Xue, J., Tejero, J., Tsang, M., Gladwin, M. T., & Corti, P. (2020). Nitrite Improves Heart Regeneration in Zebrafish. *Antioxidants & Redox Signaling*, *32*(6), 363–377. https://doi.org/10.1089/ars.2018.7687
